# Life-supporting functional kidney replacement by integration of embryonic metanephros-bladder composite tissue transplants

**DOI:** 10.1016/j.kint.2025.02.024

**Published:** 2025-03-22

**Authors:** Yoshitaka Kinoshita, Eiji Kobayashi, Kenji Matsui, Yuka Inage, Keita Morimoto, Shutaro Yamamoto, Satomi Iwai, Kento Kitada, Kentaro Iwasawa, Yatsumu Saito, Toshinari Fujimoto, Kei Matsumoto, Shushi Nagamori, Akira Nishiyama, Haruki Kume, Takanori Takebe, Takashi Yokoo, Shuichiro Yamanaka

**Affiliations:** 1 Division of Nephrology and Hypertension, Department of Internal Medicine, The Jikei University School of Medicine, Tokyo, Japan; 2 Department of Urology, Graduate School of Medicine, The University of Tokyo, Tokyo, Japan; 3 Department of Kidney Regenerative Medicine, The Jikei University School of Medicine, Tokyo, Japan; 4 Department of Pediatrics, The Jikei University School of Medicine, Tokyo, Japan; 5 Department of Urology, The Jikei University School of Medicine, Tokyo, Japan; 6 Laboratory of Small Animal Surgery 2, School of Veterinary Medicine, Kitasato University, Aomori, Japan; 7 Department of Pharmacology, Faculty of Medicine, Kagawa University, Kagawa, Japan; 8 Division of Gastroenterology, Hepatology and Nutrition, Developmental Biology and Center for Stem Cell and Organoid Medicine (CuSTOM), Cincinnati Children’s Hospital Medical Center, Ohio, USA; 9 Center for Stable Isotope Medical Research, The Jikei University School of Medicine, Tokyo, Japan; 10 Department of Pediatrics, University of Cincinnati College of Medicine, Ohio, USA; 11 Premium Research Institute for Human Metaverse Medicine (WPI-PRIMe), and Department of Genome Biology, The University of Osaka, Osaka, Japan; 12 Human Biology Research Unit, Institute of Integrated Research, Institute of Science Tokyo (Science Tokyo), Tokyo, Japan

**Keywords:** embryonic kidney, homeostasis, life-support, maturation, metanephros, transplantation

## Abstract

Novel transplantable organs need to be developed to address the global organ shortage. Transplantation of embryonic kidney tissue, or metanephros, facilitates glomerular and tubular maturation and offers partial organ functional support. However, adult environments do not permit exponential growth in size, limiting the life-supporting functionality and organ replacement effect of this approach. Here, we developed a novel strategy that combines the fusion of embryonic bladders with multiple anastomoses to the host ureter, enabling a significant increase in metanephros transplantation and urinary tract integration. By surgically anastomosing divided bladder segments, we reconstructed the excretory pathways by merging four metanephroi into each bladder and integrating them with the host ureter. Following the transplantation and integration of 20 metanephroi at the para-aortic region, anephric rats survived for over a month and generated approximately 50,000 nephrons *in vivo*. Ultrastructural and single-cell–transcriptomic analyses revealed that the maturity of the transplanted metanephroi was comparable to that of adult kidneys, although their small size likely contributed to their decreased urine concentration ability. Postoperative support helped normalize physiological homeostasis, including solute clearance, acid–base balance, electrolyte levels, and kidney hormone levels, within vital ranges. Our findings underscore the functional maturation capacity and dose-dependent therapeutic efficacy of embryonic kidney tissue, suggesting its potential as a transplantable organ system.

Efforts to address the imbalance between the number of patients requiring organ transplantation and the limited availability of donors have led to extensive research into the development of novel transplantable organs. Despite significant advancements in xenotransplantation^1,2^ and organoid technology,^3–6^ creating a fully functioning organ possessing a complex 3-dimensional structure while also maintaining low immunogenicity remains challenging. In parallel, researchers have explored the use of immature embryonic tissues as viable transplantation sources because of their potential to mature into chimeric structures on host vasculature infiltration.^7,8^ This approach offers immunological advantages over mature organs^9–11^ and presents avenues for organ humanization strategies by leveraging developmental processes,^12,13^ as well as strategies to treat fetal organ failure by utilizing their small size.^14^

In the context of kidneys, transplantation of metanephros (MN), the embryonic kidney primordia, into adult hosts has reportedly produced urine-like fluid,^7,8^ which can be drained outside the body after reconstructing the urinary tract.^15^ However, MN transplants result in limited growth in size compared to adult kidneys, offering only marginal prolongation of survival compared with that in anephric conditions.^15–17^ We hypothesized that transplanting multiple MNs would significantly prolong the survival of anephric subjects. However, the technical complexity of reconstructing the urinary tract presents a significant obstacle in scaling up transplant procedures.^17^ This barrier impedes efforts to elucidate the factors preventing complete kidney function replacement using this approach—whether attributed to an insufficient tissue volume, inadequate cellular or microtissue maturity, or a combination thereof.

Here, we established a novel strategy for transplanting and integrating a substantial number of embryonic kidneys into the urinary tract of the host. The bladder segments of the metanephros–bladder composites (MNBs) were divided and surgically anastomosed to facilitate the simultaneous growth of 4 MNs per each fused bladder without inducing hydronephrosis. All fused bladders were subsequently connected to the host ureter, enabling the transplantation and integration of up to 20 MNs.

We aimed to replace kidney function in a rat model by transplanting a substantial number of embryonic kidneys, demonstrating the proof of concept that embryonic organs can attain functional maturity *in vivo* and that organ function can be replaced by integrating sufficient tissues into the host organ system. Our findings highlight the potential of embryonic organ transplantation as a therapeutic strategy to address organ shortage.

## METHODS

### Experimental animals and ethics

All animal experiments were approved by the Jikei Medical University Animal Research Ethics Committee (approval number 2021–069). Adult LEW/SsNSlc (LEW) rats (male, 8 weeks old) and pregnant LEW and SD-Tg (CAG-EGFP) rats (16th–17th days of pregnancy) were obtained from Nihon SLC. Adult NOD/ShiJic-scid Jcl (NOG) mice (male, 6 weeks old) were obtained from CLEA Japan. The animals were housed in cages under controlled conditions, including temperature (20–24 °C), humidity (45%–65%), and a 12-hour light/dark cycle, and provided with standard chow (CE-2, CLEA Japan) and water *ad libitum*, unless specified otherwise. All animal handling procedures adhered to the guidelines and regulations of the institution.

### Integrated MNB grafts

**Figure 1 |:**
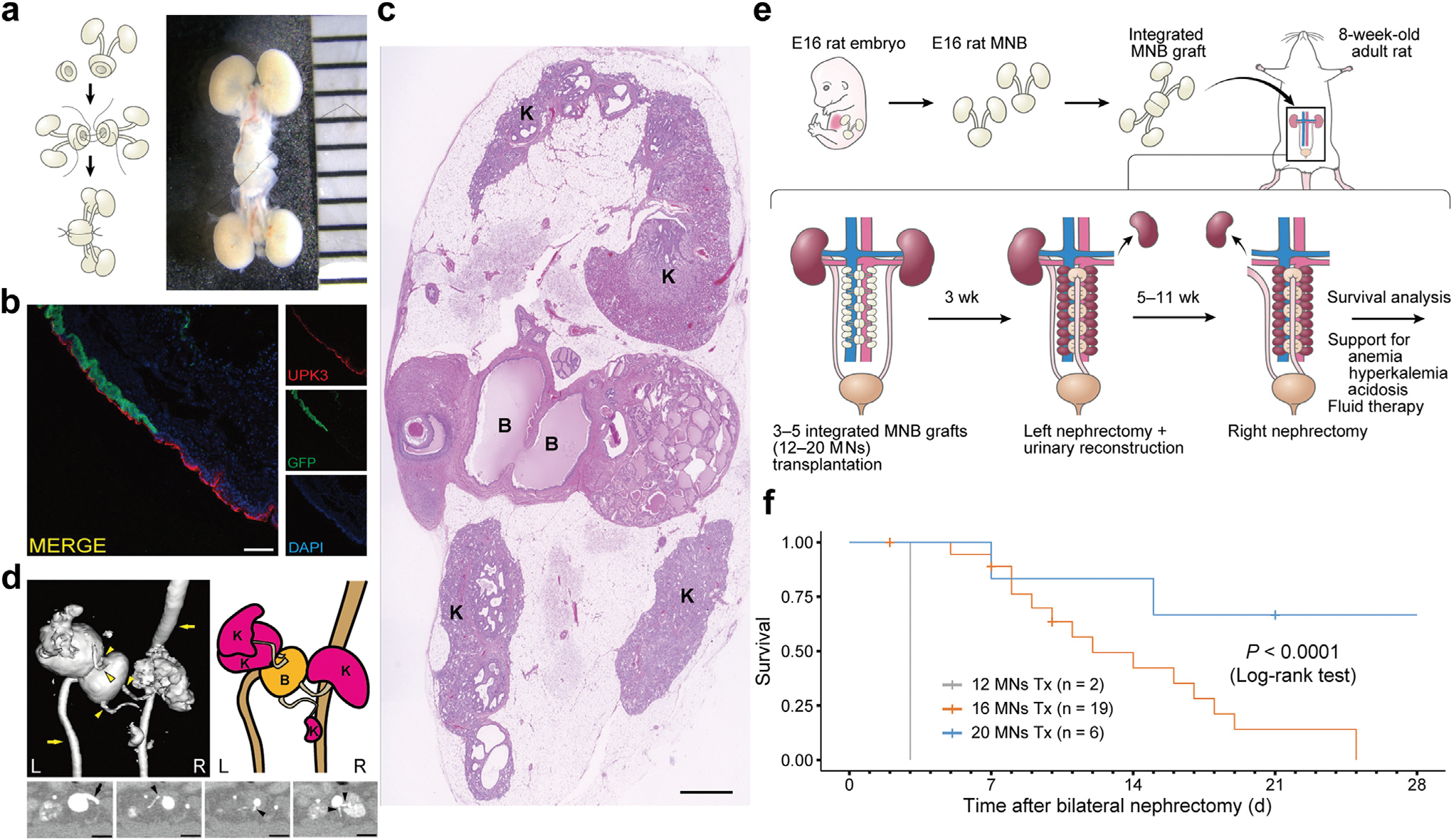
Dosage effect of integrated metanephros–bladder composite (MNB) transplantation. (**a**) Schematic (left) and a representative image (right) of an integrated MNB graft. The bladder segment of the embryonic day 16 (E16) MNB is divided, and the anterior and posterior walls are sutured. Each tick on the background represents 1 mm. (**b**) Immunofluorescence of the fused bladder of integrated MNB grafts transplanted (Tx) into immunocompromised mice at 3 weeks post-transplantation, derived from green fluorescent protein (GFP)–positive and –negative rat MNBs. GFP-positive and -negative cells form a contiguous urothelial barrier stained with uroplakin III and share the bladder lumen. Bar = 100 μm. (**c**) Hematoxylin and eosin staining of integrated MNB grafts 5 weeks after anastomosing to the host ureter at 3 weeks post-transplantation into syngeneic rats. Four transplanted kidneys (K) with no hydronephrosis surround the fused bladder (B). Bar = 1 mm. (**d**) Three-dimensional reconstructed images (left [L]), schematic representation (right [R]), and axial images (bottom) of contrast-enhanced computed tomography to visualize the urinary tract. Urine from 4 transplanted kidneys converges into the fused bladder via the transplanted ureters (arrowhead), with excretion observed from the host ureters (arrow). The axial images highlight the junction where the host ureter (left) and the 4 transplanted embryonic ureters (second, third, and fourth from the left) connect to the fused bladder. Bars = 5 mm. (**e**) Experimental workflow of multiple integrated MNB graft transplantation, urinary tract reconstruction, host kidney removal, and survival analysis in a syngeneic rat model. (**f**) Kaplan–Meier curve showing the survival of rats transplanted with 12, 16, and 20 metanephros (MNs). Euthanasia for sample collection and deaths attributed to technical errors during anesthesia were censored. To optimize viewing of this image, please see the online version of this article at www.kidney-international.org.

Following cesarean section delivery of embryonic days 16 and 17 fetal LEW rats via an abdominal midline incision, immediate euthanasia of both fetuses and pregnant rats was conducted via decapitation and exsanguination, respectively. MNBs were procured *en bloc*, as previously described.^15^ The bladder of each MNB was divided, and the anterior and posterior walls were sutured with an 11–0 nylon thread (Figure 1a). For experiments involving confirmation of bladder fusion, fetuses were obtained from SD-Tg (CAG-EGFP) pregnant rats, and integrated MNB grafts were prepared from MNBs of fetuses with and without transgene, as determined by the presence or absence of green fluorescence.

### Transplantation and urinary reconstruction

Rat MNBs or integrated MNB grafts were transplanted into the retroperitoneal cavities of adult NOG mice or LEW rats, and tissue retrieval or urinary tract reconstruction was performed 3 weeks post-transplantation. For urinary tract reconstruction, the left native kidney was removed, and the left ureter was sectioned at the level of the renal hilum and anastomosed to the graft bladders to enable the drainage of transplant-derived urine from the body.

### Survival analysis

Rats receiving transplantation and reconstruction with multiple integrated MNB grafts had their remaining right kidney removed 8 to 14 weeks post-transplantation. Postoperatively, body weight and water consumption were measured once daily, along with assessments of their condition. Blood samples were collected from the tail vein daily during the first post-operative week and twice a week subsequently. Simultaneously, 24-hour urine samples were collected once a week using a metabolic cage (KN-650-MC-ST, Sugiyama-gen). Animals received treatment for complications associated with low-level kidney function and subcutaneous supplemental fluid therapy as deemed necessary by the observers based on body weight and blood test results (Supplementary Table S1).^18^ For survival analysis with water intake enhancement, postoperative management was conducted using established protocols (Supplementary Figure S1, Supplementary Table S2). To encourage water intake and recovery from dehydration, 5% glucose was added to the drinking water, and high-caloric, water-rich gel supplement (DietGel Recovery, Clear H_2_O) was administered. Given that chronic kidney disease model animals typically exhibit severe weight loss from baseline,^19,20^ a weight loss of 30% from the time of nephrectomy was set as the euthanasia criterion.^21^ Additionally, regardless of body weight, the inability to ambulate was considered a moribund condition, prompting euthanasia by exsanguination under general anesthesia. Survival time was defined based on the last day the animals remained alive and healthy. Sham-operated rats underwent identical procedures and postoperative protocols except for MNB transplantation and urinary tract reconstruction.

### Immunohistochemistry

The details are described in the Supplementary Methods and Supplementary Table S3.

### Electron microscopy

The details are described in the Supplementary Methods.

### Quantitative real-time polymerase chain reaction

The details are described in the Supplementary Methods and Supplementary Table S4.

### scRNA-seq of transplanted embryonic kidneys

Single-cell RNA-sequencing (scRNA-seq) data of kidney samples from healthy rats at postnatal days 0, 2, 5, 10, 20, and 56^22^ and of transplanted MN samples at 3 weeks after host kidney removal (16 weeks post-transplantation) were analyzed. Details of the methods are included in the Supplementary Methods.

### Sodium, potassium, and urea contents in the renal cortex and medulla

Kidney samples were dried at 90 °C for 72 hours, and the dry weight was determined. The samples were ashed at 450 °C for 36 hours and 600 °C for 12 hours. Next, the samples were dissolved in 5 mL of 10% HNO_3_, and the sodium and potassium levels were measured using a flame photometer (ANA-135; Tokyo Photoelectric). Additionally, the renal urea content was measured in different samples using a commercially available kit (Abcam), as described previously.^23,24^

### Quantification of the length of thin descending limb segments

To estimate the length of AQP1-positive thin descending limb segments, immunostained sections of kidney maximum cross-sections were captured using a fluorescence microscope (BZ-X800, Keyence). Subsequently, the number of AQP1-positive tubular cross-sections and PDGFRB-positive glomerular cross-sections were manually counted.^25^

### Urine concentration test

To examine urine-concentrating ability, we collected urine during both 24-hour *ad libitum* feeding and 16-hour fasting periods^26^ using metabolic cages and assessed urine volume and osmolarity. These collections were conducted under a low-potassium, 2% sodium bicarbonate-added diet, 5% glucose drinking water, and DietGel Recovery supplementation.

### Statistical analyses

The sample size for survival analysis following established protocols was determined by assuming a mean survival time of 2 days for the control group and 28 days for the transplant group.^27,28^ This calculation aimed to achieve a detection power of 0.8 with an α error of 0.05 using the log-rank test. Data visualization and statistical analyses were conducted using R version 4.2.1 software (R Foundation for Statistical Computing).^29^ Intergroup comparisons were conducted using Student’s unpaired *t* test, whereas survival analysis was performed using the Kaplan–Meier method to plot survival curves, followed by log-rank testing. A 2-tailed *P* value <0.05 was considered statistically significant.

## RESULTS

### Increased MNB grafts enabled the survival of anephric rats

Despite their ability to produce urine-like fluid on *in vivo* transplantation, embryonic kidneys have not been associated with long-term survival in host organisms.^15–17^ To investigate the effect of transplanting an increased volume of tissues to replace kidney function in a rat model, we modified a previously reported *en bloc* MNB transplantation technique.^15^ Specifically, bladder segments from MNBs were divided and surgically anastomosed to form an integrated MNB graft (Figure 1a). On *in vivo* transplantation, this approach resulted in the fusion of bladders, enabling the sharing of luminal space (Figure 1b), and thereby preventing hydronephrosis in all 4 MNs after connection to the host ureter (Figure 1c, Supplementary Figure S2). The success rate of urinary tract connection improved from 66.7% (6 of 9) with single MNBs to 83.3% (10 of 12) with integrated MNB grafts, likely due to increased urine accumulation in the fused bladders (Supplementary Figure S3). Contrast-enhanced computed tomography revealed a urinary tract connection from the four MNs to the fused bladder, continuing to the host ureter and finally to the host bladder (Figure 1d, Supplementary Movie S1). Next, the multiple integrated MNB grafts were transplanted onto the anterior segment of the abdominal aorta. Left nephrectomy and urinary tract reconstruction were performed 3 weeks post-transplantation, followed by right nephrectomy at 8 to 14 weeks post-transplantation for survival analysis of syngeneic recipient rats (Figure 1e). The success rates of urinary tract connection in rats transplanted with 3, 4, and 5 integrated MNB grafts were 100% (2 of 2), 87.0% (20 of 23), and 92.9% (13 of 14), respectively, including cases not used for survival analysis. In total 39 rats, only 1 surgery-related death (2.6%) occurred due to anesthesia during urinary tract reconstruction. Following nephrectomy, appropriate supportive therapies were administered to manage complications associated with low-level kidney function, such as acidosis, renal anemia, and hyperkalemia, as well as fluid replacement for dehydration, as necessary.^18^ Rats transplanted with 12 MNs did not survive beyond 1 week. However, survival periods were positively correlated with the number of transplants; rats transplanted with 20 MNs achieved long-term survival exceeding 1 month (Figure 1f, Supplementary Figure S4, and Supplementary Table S1). These findings indicate that the barrier to renal function replacement for sustaining life was overcome by increasing the volume of transplanted tissues and ensuring full integration with the host urinary tract.

### Gene expression profiles in MN grafts resembled those of adult kidneys

**Figure 2 |:**
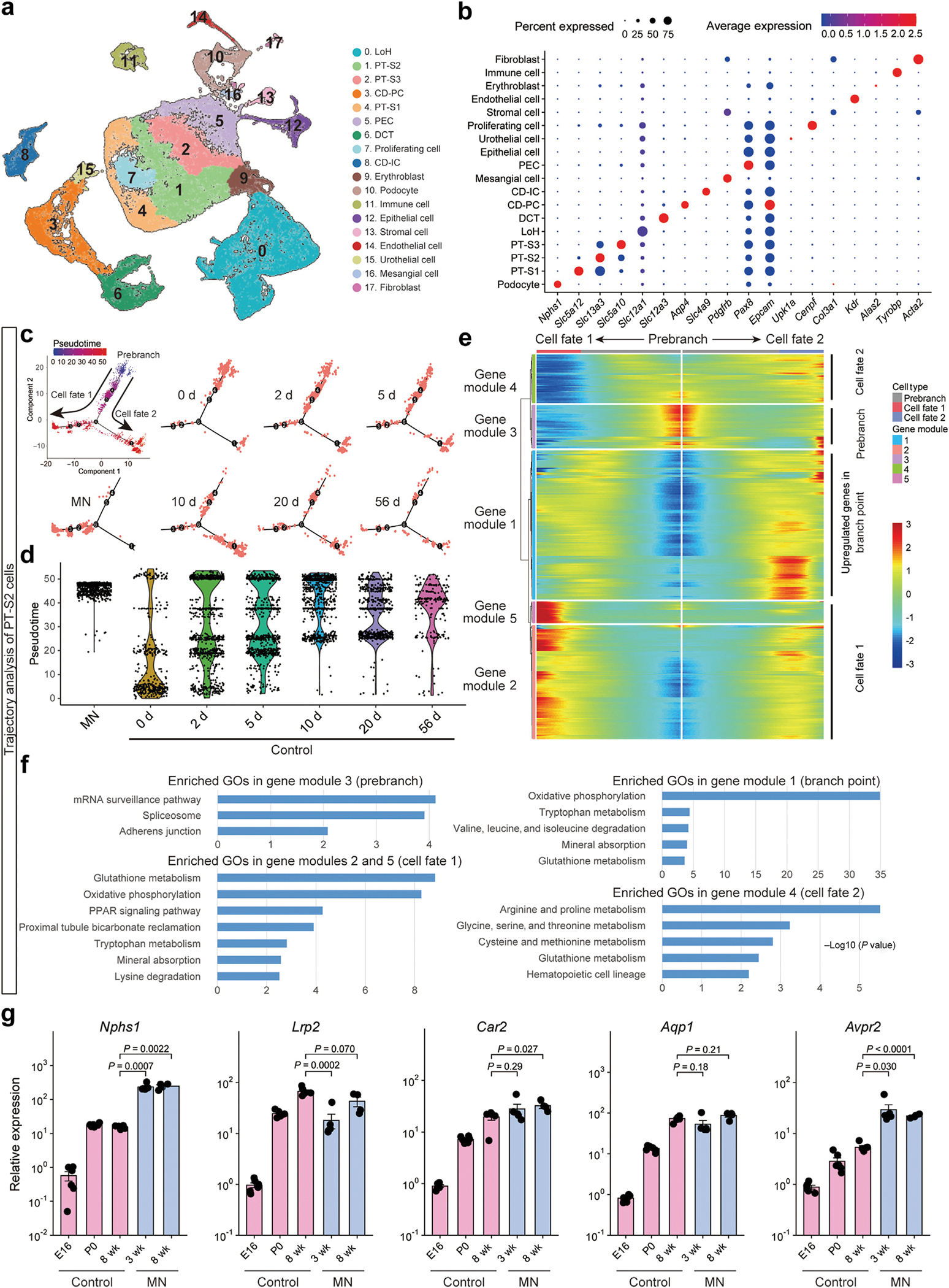
Transcriptomic profiling of transplanted metanephros (MN) grafts and adult kidneys. Single-cell RNA-sequencing (scRNA-seq) data from transplanted MNs sampled 3 weeks after bilateral nephrectomy (16 weeks after integrated MNB transplantation) were analyzed after integration with scRNA-seq data obtained from kidney samples at postnatal days 0 (P0), 2, 5, 10, 20, and 56. (**a**) Uniform manifold approximation and projection displaying unsupervised clustering of all 53,994 cells into 18 cell types with annotations based on the expression of established marker genes. (**b**) Dot plot showing the expression levels of representative marker genes in the 18 clusters. (**c**) *t*-Distributed stochastic neighbor embedding (t-SNE) plot showing Monocle 2-based trajectory analysis of proximal tubule segment 2 (PT-S2) cells, the cluster with the highest cell count in the MN sample. Cells are colored based on the predicted pseudotime. (**d**) Violin plot showing pseudotime distribution of PT-S2 cells from MN sample and healthy controls at various ages (P0–P56). Most cells derived from the MN samples show advanced pseudotime. (**e**) Heatmap depicting branch-dependent genes during the transition from pseudotime-young to pseudotime-old cells along the trajectory timeline. Five gene modules associated with branches in t-SNE were identified. Cells from the MN samples were predominantly observed in the cell fate 1 branch, showing upregulation in gene modules 2 and 5. (**f**) Gene ontologies (GOs) enriched in gene modules corresponding to each state along the trajectory: module 3 to prebranch state, module 1 to branch point, modules 2 and 5 to cell fate 1, and module 4 to cell fate 2, respectively. (**g**) Quantitative real-time polymerase chain reaction analysis of maturation-related markers in various nephron segments. Gene expression was compared among embryonic day 16 (E16), P0, and 8-week control kidneys, and MNs at 3 weeks post-transplantation (without urinary tract reconstruction) and 8 weeks post-transplantation (5 weeks after urinary tract reconstruction). Expression values are normalized to *Gapdh* and shown relative to one of the E16 control samples. Data are mean ± SEM. Points represent biological replicates from at least 4 independent experiments. Statistical significance was determined by 2-tailed Student’s unpaired *t* tests. CD-IC, intercalated cell of the collecting duct; CD-PC, principal cell of the collecting duct; DCT, distal convoluted tubule; LoH, Loop of Henle; PEC, glomerular parietal epithelial cell.

To evaluate the cellular maturity of the transplanted MN grafts, we conducted a comparative analysis of gene expression in kidney samples from healthy rats at postnatal days 0, 2, 5, 10, 20, and 56^22^ and transplanted MN samples collected at 16 weeks post-transplantation (3 weeks after host kidney removal at week 13) using scRNA-seq. Using previously reported marker genes,^30–32^ various renal cell types were identified (Figure 2a and b); although certain cell populations, including loops of Henle and podocytes, showed relatively lower abundance in MN samples, MN-derived cells exhibited a comprehensive coverage of cell types with a composition comparable to control kidneys (Supplementary Figure S5). Trajectory analysis was conducted for each renal cell cluster^33,34^ and revealed a lining along the pseudotime from neonatal to adult kidney cells, indicating the maturation process (Figure 2c and d, Supplementary Figure S6). MN sample was characterized by cells at advanced pseudotime states, suggesting that their gene expression patterns were similar to those of adult controls. For proximal tubule segment 2 cells, selected for their high abundance across all cell types in the MN sample, we performed gene ontology analysis of differentially expressed genes at each state along the trajectory (Figure 2e and f). Comparison of enriched gene ontologies revealed developmental transitions: the prebranch state showed immature transcriptional features such as protein synthesis and cellular adhesion, while the branch point and subsequent cell fates were enriched in maturation-associated pathways, including energy production and amino acid metabolism.^35,36^ While both cell fates maintained these maturation signatures, the MN cells were predominantly distributed to cell fate 1, which showed enrichment in PPAR signaling, known to mediate compensatory hypertrophy after nephron loss,^37^ and tubule function-associated pathways.^38^ This distinct distribution pattern likely reflects functional adaptation of MN tissue to the uremic environment after host nephrectomy. To further evaluate the maturation status across all nephron segments, we performed quantitative real-time polymerase chain reaction analysis of kidney tissue. The expression levels of maturation markers for various nephron segments^39–42^ in MNs at 8 weeks post-transplantation were comparable to or higher than those in adult control kidneys (Figure 2g, Supplementary Figure S7). These findings suggest that the transplanted MNs achieved gene expression patterns largely comparable to those of adult kidneys.

### MN grafts showed gradual progression to structural and functional maturity

**Figure 3 |:**
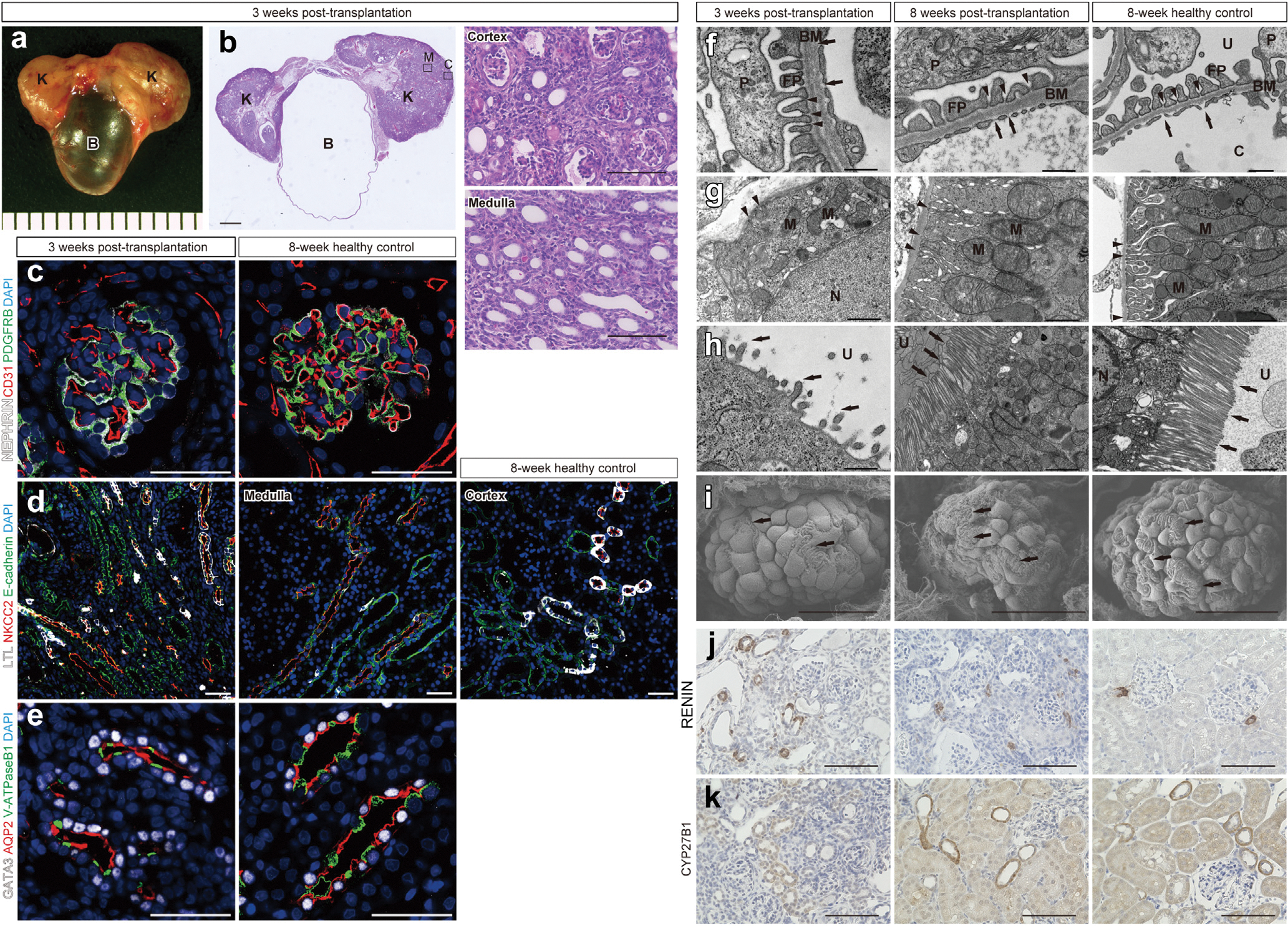
Longitudinal structural and functional maturation of metanephros (MN) grafts. (**a**) Metanephros-bladder composite (MNB) 3 weeks post-transplantation (before urinary reconstruction). Each tick on the background represents 1 mm. (**b**) Hematoxylin and eosin staining of MNB 3 weeks post-transplantation into syngeneic rats. Two kidneys (K) have developed adjacent to the bladder, filled with urine (B). Higher magnification images of cortex and medulla are shown on the right (corresponding to the regions marked C and M in the left panel). Bars = 1 mm (left) and 100 μm (right). (**c–e**) Immunofluorescence of MNs (left) and 8-week healthy control kidneys (right). Bars = 50 μm. (**c**) Glomerular structures supported by PDGFRB-positive mesangial cells, nephrin-positive podocytes, and cluster of differentiation 31 (CD31)–positive endothelial cells. (**d**) Tubular segments, including lotus tetragonolobus lectin (LTL)–positive proximal tubules, sodium-potassium-chloride cotransporter 2 (NKCC2)–positive loops of Henle, and E-cadherin-positive distal tubules. (**e**) Collecting ducts formed by AQP2-positive principal cells and vacuolar-type ATPase B1 (V-ATPaseB1)–positive intercalated cells. Nuclei of the collecting ducts stained with GATA3. (**f–k**) Ultrastructural observation and immunohistochemistry of MN samples at 3 weeks post-transplantation (before urinary reconstruction), 8 weeks post-transplantation (5 weeks after urinary reconstruction), and 8-week healthy control kidneys. (**f–h**) Transmission electron microscopy images of glomeruli (f) and the basolateral (**g**) and luminal (h) sides of proximal tubular cells. (f) Endothelial cells supported by the 3-layered glomerular basement membrane (BM), along with podocytes (P) and foot processes (FPs). Slit diaphragms (arrowheads) are visible between FPs. Arrows represent the fenestrae of endothelial cells. Bar = 500 nm. (**g,h**) At 3 weeks post-transplantation, microvilli (arrows) are sparse and short, with mitochondria (M) present but showing limited basolateral infoldings (arrowheads). By 8 weeks post-transplantation, microvilli (arrows) are extended, and mitochondria are aligned along the developed basolateral infoldings (arrowheads). Bars =− 1 μm (**g**), 500 nm (**h**, left) and 2 μm (**h**, middle and right). (**i**) Scanning electron microscopy images of glomeruli. At 3 weeks post-transplantation, interdigitation of FPs (arrows) extending from podocytes can be observed; however, it is segmental and not well-developed in some areas. By 8 weeks post-transplantation, it developed prominently in the glomerulus. Bars = 20 nm (left), 30 nm (middle), and 50 nm (right). (**j,k**) Immunohistochemistry of renin and CYP27B1. Bars = 100 μm. (**j**) Renin-producing cells were present in the arterial wall apart from the glomerulus at 3 weeks post-transplantation but gradually assemble in the juxtaglomerular apparatus by 8 weeks post-transplantation. (**k**) CYP27B1 expression has gradually strengthened and reached a comparable level to that in adults by 8 weeks post-transplantation. C, capillary; DAPI, 4′,6-diamidino-2-phenylindole; N, nucleus; U, urinary space (Bowman’s space). To optimize viewing of this image, please see the online version of this article at www.kidney-international.org.

Histologic and ultrastructural evaluations were performed longitudinally to understand the process of functional maturation of MN grafts. MNBs transplanted into syngeneic rats developed into 2 kidneys connected to the bladder filled with urine at 3 weeks post-transplantation (Figure 3a and b). Immunostaining indicated the growth of various nephron segments, including the glomeruli, tubules, and collecting ducts (Figure 3c–e). However, electron microscopy at this stage revealed segmental interdigitation of podocyte foot processes, short microvilli, and minimal basolateral infoldings of the proximal tubules, indicating immature ultrastructural characteristics (Figure 3f–i, left).^43^ At 8 weeks post-transplantation, interdigitation of foot processes had become widespread throughout the glomeruli, microvilli had elongated, and basolateral infoldings had increased in the proximal tubules (Figure 3f–i, middle), indicating ultrastructural maturity comparable to that of adult kidneys (Figure 3f–i, right). Immunostaining of kidney-derived hormones revealed that renin-producing cells gradually assembled in the juxtaglomerular apparatus^44–46^ by 8 weeks post-transplantation (Figure 3j); the expression of CYP27B1, a vitamin D hydroxylase, also increased to a level comparable to that in adults^47^ by 8 weeks post-transplantation (Figure 3k). Based on these findings, we confirmed that the basic kidney structure was present with initiation of urine production within the first 3 weeks post-transplantation. However, it should be noted that ultrastructural maturation and development of functional capabilities of the grafts, including hormone production, continued and reached comparable levels to those in adult kidneys by 8 weeks post-transplantation.

### Host-derived vessel infiltration established functional vascular networks within MN grafts

**Figure 4 |:**
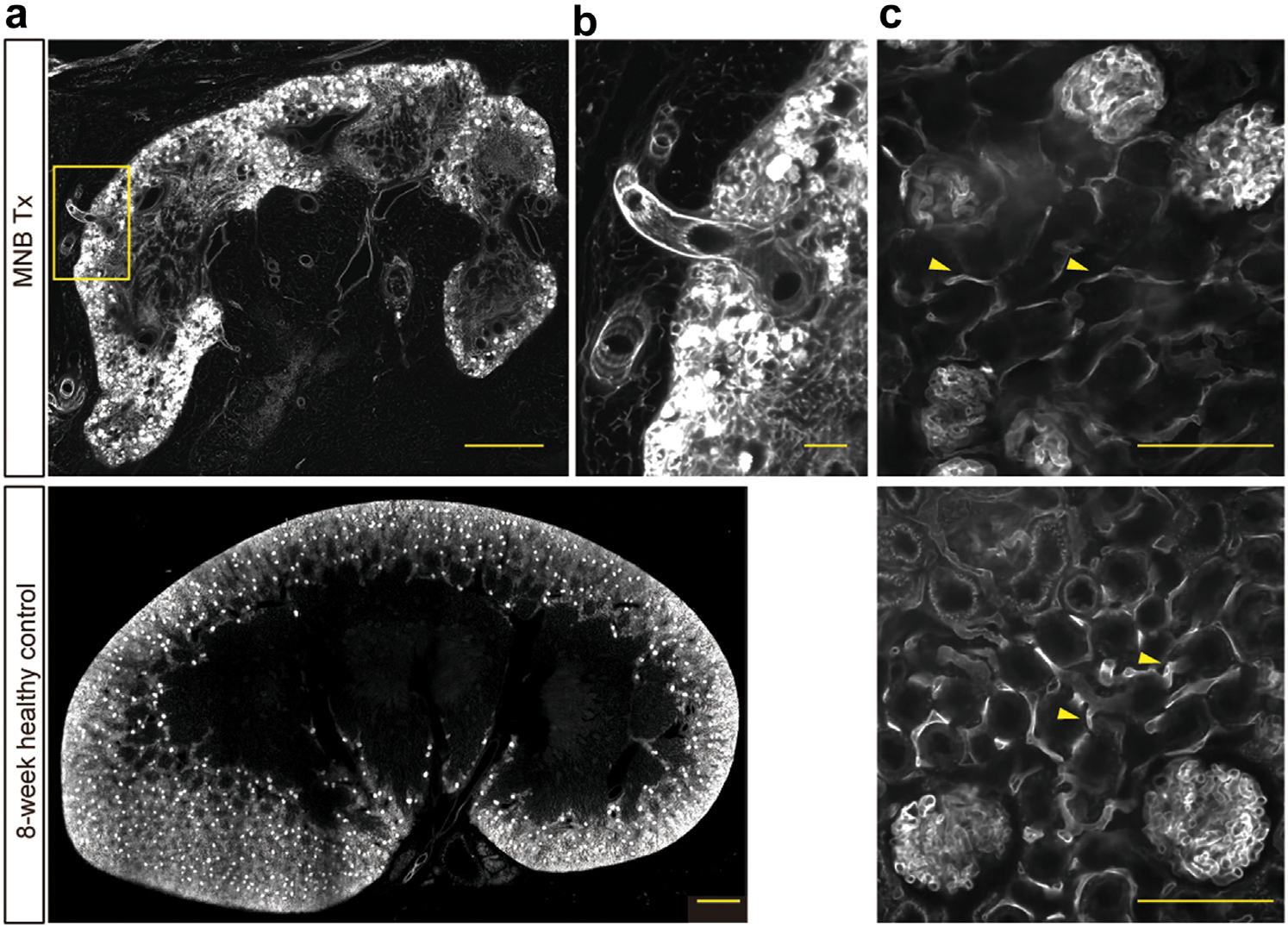
Confirmation of blood flow and visualization of vasculature within metanephros (MN) grafts. Visualization of blood vessels in MN grafts at 13 weeks post-transplantation and 8-week healthy control kidneys using fluorescently labeled lectin perfusion and tissue clearing. (**a**) Global vascular networks of MN graft and control kidney. Distinct spots of lectin accumulation represent glomeruli. Yellow box indicates the region shown in (**b**). Bars = 1 mm. (**b**) Blood vessels infiltrating from the capsular region of MN grafts. Bar = 100 μm. (**c**) Higher magnification images showing well-developed glomerular capillaries and peritubular capillaries (arrowheads). Bars = 100 μm. MNB, metanephros–bladder composite; Tx, transplanted grafts. To optimize viewing of this image, please see the online version of this article at www.kidney-international.org.

To analyze vascular development within MN grafts, we visualized capillary networks by tissue clearing of grafts after intravenous injection of fluorescently labeled lectin that binds to vascular endothelium (Figure 4a, Supplementary Movie S2).^48^ Unlike native kidneys where blood supply is delivered through a single renal artery, the host vessels appeared to infiltrate from the surrounding tissues including the capsular region (Figure 4b). Within the grafts, well-developed glomerular capillaries and peritubular capillaries were formed, exhibiting functional connections with the host vasculature (Figure 4c, Supplementary Movie S3). These findings demonstrated that transplanted MN grafts recruited surrounding vessels and established the vascular networks necessary for their function.

### Limited MN size impaired urine concentration ability

**Figure 5 |:**
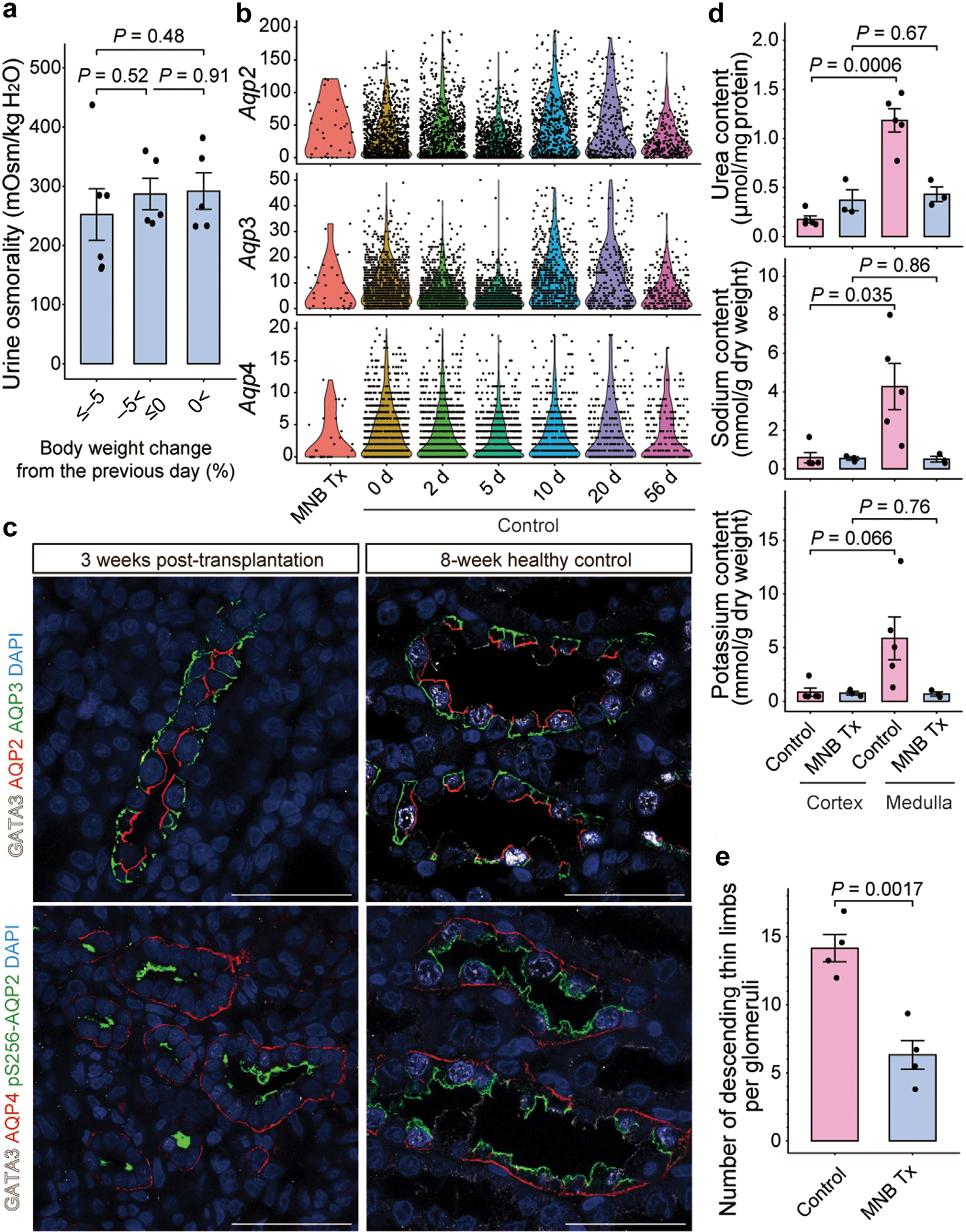
Small kidney size impairs urine-concentrating ability. (**a**) Osmolality of urine collected overnight using metabolic cages during *ad libitum* water and food intake. Despite the decrease in body weight from the previous day, the rise in urine osmolality is minimal. (**b**) Violin plots showing the expression of *Aqp2*, *Aqp3*, and *Aqp4* genes in the principal cells of the collecting ducts in transplanted kidneys (metanephros–bladder composite [MNB] Tx) and healthy rat kidneys at postnatal days 0, 2, 5, 10, 20, and 56 (control) obtained from single-cell RNA-sequencing data. Comparable expression levels are observed in transplanted kidneys and healthy controls. (**c**) Immunofluorescence showing localization of each aquaporin protein in collecting duct principal cells of MNs at 3 weeks post-transplantation (left) and 8-week healthy control kidneys (right). Bars = 50 μm. (**d**) Tissue concentrations of urea, sodium, and potassium in the cortex and medulla of the embryonic kidneys at least 8 weeks post-transplantation (MNB Tx) and in the kidneys of healthy 8-week-old rats (controls). Medullary osmolyte concentration has increased compared with that in the cortex in the controls; however, this increase is limited in the transplanted MNs. (**e**) Number of AQP1-positive thin descending limb cross-sections per glomerulus, as quantified via tissue immunostaining. To estimate the length of AQP1-positive segments, AQP1-positive tubular and PDGFRB-positive glomerular cross-sections were counted in the maximum cross-sectional slice and subsequently divided. (**a,d,e**) Data are mean ± SEM. Points are biological replicates examined over at least 3 independent experiments. Statistical analyses were performed using 2-tailed Student’s unpaired *t* tests. DAPI, 4′,6-diamidino-2-phenylindole. To optimize viewing of this image, please see the online version of this article at www.kidney-international.org.

Although transplanted kidneys exhibit histologic and transcriptional features similar to those of adult kidneys, we observed episodes of dehydration potentially attributed to polyuria in MNB-transplanted rats. Urine osmolality analysis showed a minimal increase despite weight loss (Figure 5a). Urine concentration occurs through vasopressin-dependent passive absorption of water molecules from the primary urine in the collecting ducts into the hyperosmotic medullary environment established by the countercurrent multiplier system of the loops of Henle.^49,50^ Transcripts of the aquaporin channels, which regulate water movement in the collecting ducts, were expressed at levels nearly equivalent to those in healthy rat kidneys (Figure 5b). Immunostaining of collecting duct principal cells demonstrated that AQP3 and AQP4 were properly localized to the basolateral membrane,^51^ while AQP2 was phosphorylated and localized to the apical membrane, suggesting intact vasopressin signaling (Figure 5c).^52^ In contrast, tissue concentrations of osmolytes were observed to be lower in the transplanted kidney medulla (Figure 5d). The length of AQP1-positive descending thin limbs, which contribute to the formation of osmotic gradients,^53^ was observed to be shortened (Figure 5e). Therefore, the length of the countercurrent multiplier system may be insufficient, leading to impaired formation of the medullary hyperosmotic environment and decreased urine-concentrating ability.^54^ These findings suggest that suboptimal organ size may impede optimal functionality despite the achievement of sufficient maturation at the cellular and microstructural levels.

### Optimized management enabled stable homeostasis in MN graft recipients

**Figure 6|:**
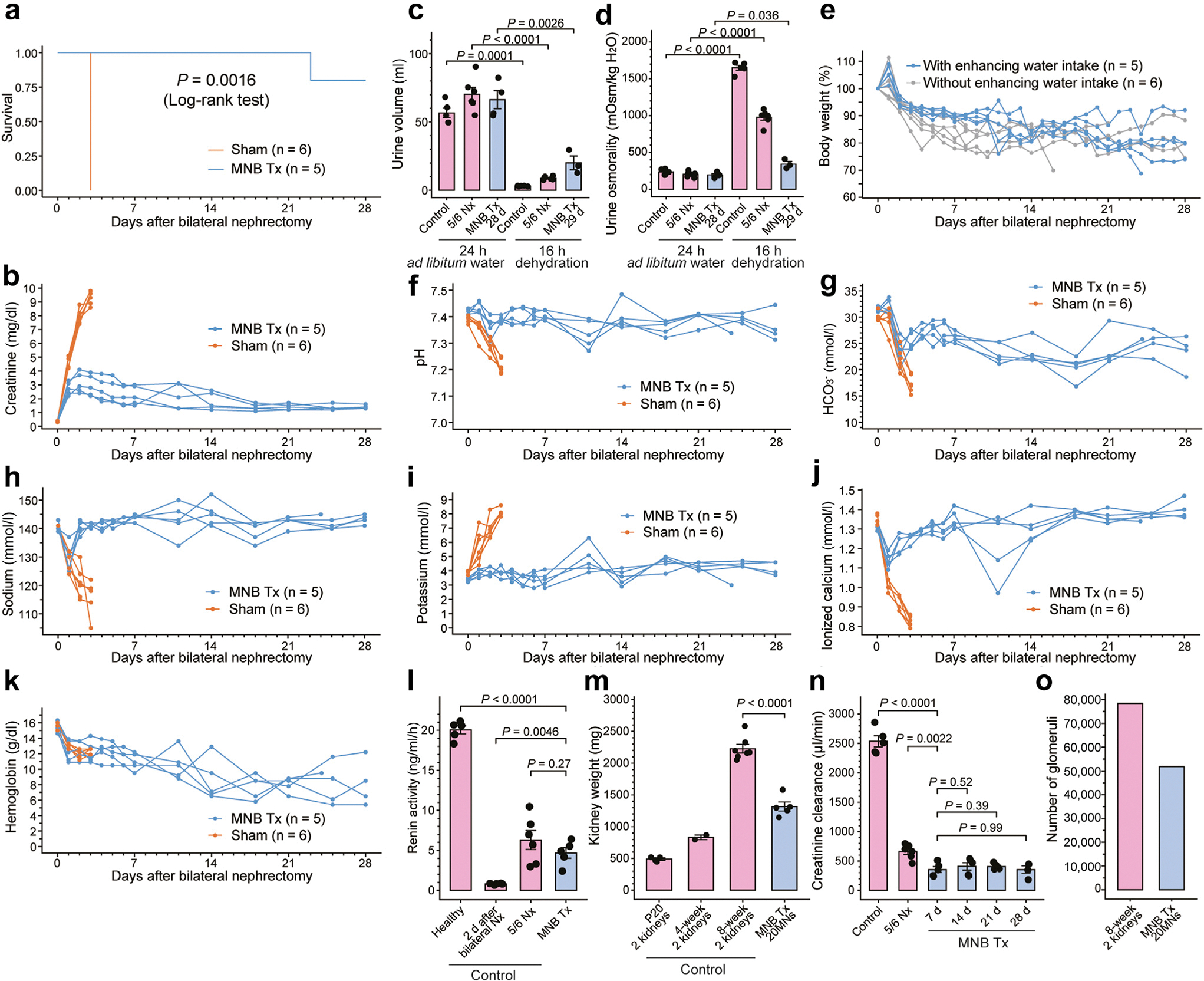
Renal functional assessment after refined postoperative management. (**a,b**) Kaplan–Meier curve showing the survival (**a**) and changes in serum creatinine levels (**b**) in 5-integrated metanephros-bladder composite graft-transplanted rats (MNB Tx) and sham-operated (sham) rats. (**c,d**) Urine volume (**c**) and osmolality (**d**) of 5-integrated MNB Tx rats and healthy 8-week-old and 5/6 nephrectomy (5/6 Nx) rats (control). Urine during a 24-hour period with *ad libitum* access to food and water and a 16-hour fasting period was collected using metabolic cages. (**e**) Changes in body weight of 5-integrated MNB Tx rats with or without water intake enhancement. Data were normalized to the body weight on the day of host kidney removal. Early postoperative weight loss was alleviated through water intake enhancement. (**f–k**) Changes in blood levels of pH (**f**), bicarbonate (HCO_3_^−^) (**g**), sodium (**h**), potassium (**i**), ionized calcium (**j**), and hemoglobin (**k**) in 5-integrated MNB Tx rats and sham rats. (**l**) Renin activity of 5-integrated MNB Tx rats and 8-week-old healthy, anephric (2 days after kidney removal), and 5/6 Nx rats (control). (**m**) Weights of 20 transplanted embryonic kidneys (MNB Tx) and bilateral kidneys of 20-day-, 4-week-, and 8-week-old healthy rats (control). (**n**) The 24-hour creatinine clearance levels of 5-integrated MNB Tx rats at 7, 14, 21, and 28 days after host kidney removal (MNB Tx) and those of healthy 8-week-old and 5/6 Nx rats (control). (**o**) Total number of glomeruli counted as distinct spherical structures, attributed to lectin accumulation in cleared tissues. Twenty metanephros (MNs) contain approximately 50,000 glomeruli, which is 60% of the glomeruli of bilateral healthy kidneys. (**c,d,l–n**) Data are mean ± SEM. Points are biological replicates examined over at least 3 independent experiments. Statistical analyses were performed using 2-tailed Student’s unpaired t tests. P20, postnatal day 20.

We next explored hydration strategies to enhance free water intake by supplementing drinking water with 5% glucose^55^ and providing a high-caloric, water-rich gel^56^ alongside the standard diet. Additionally, we standardized transplant graft conditions and established a protocol for postoperative support and fluid therapy (Supplementary Figure S1). Subsequently, further survival analysis and detailed assessments of renal functional phenotypes were performed. Among the 5 rats transplanted with 20 MNs followed by urinary tract reconstruction at 3 weeks and host kidney removal at 8 weeks, 4 exhibited 1-month survival, which was significantly longer than that observed in the sham-operated rats subjected to identical treatment except for MNB transplantation (Figure 6a). Serum creatinine levels continued to rise following host kidney removal; all sham-operated rats survived only until day 3, whereas creatinine levels peaked at days 2 and 3 postoperatively, then decreased in all MNB transplantation rats (Figure 6b). Even under conditions of induced dehydration through fasting,^26^ only a mild reduction in urine volume and a slight increase in urine osmolality were observed (Figure 6c and d). However, early postoperative weight loss was notably alleviated by the augmented water intake (Figure 6e). Stable homeostasis within vital ranges for acid–base balance and electrolyte and hemoglobin levels was achieved (Figure 6f–k) with comprehensive support. Renin activity in the systemic circulation (Figure 6l) was confirmed. The kidney weights of rats transplanted with 20 MNs were approximately 60% of the bilateral kidney weights in the 8-week-old control rats (Figure 6m), and creatinine clearance, an indicator of glomerular filtration rate, was approximately 15% (Figure 6n). The total number of glomeruli counted through tissue clearing was approximately 50,000, which is 60% of that observed in the 8-week-old control kidneys (Figure 6o, Supplementary Movie S3). These findings suggest that the transplantation of 20 embryonic kidneys provided rats with a sufficient number of nephrons and renal function for survival, enabling them to maintain fluid homeostasis and achieve sufficient survival rates with appropriate support.

## DISCUSSION

Here, we demonstrated that transplanted embryonic kidneys could replace the host kidney function by facilitating *in vivo* maturation and integrating a sufficient volume of tissue into the host organ system, thereby enabling the survival of adult rats without their native kidneys.

Regarding the maturation of transplanted MNs, while their capacity to differentiate into functional glomeruli and tubules capable of producing urine has been known,^7,57^ previous studies reported that transplanted embryonic kidneys only reached the transcriptional maturity equivalent to newborn kidneys without urinary tract reconstruction.^58,59^ Our comprehensive trajectory analysis, including ultrastructural observations, showed that further maturation proceeded after the initiation of urine production under appropriate urine drainage. Although this maturation process required a minimum of 8 weeks, our scRNA-seq analysis revealed that they eventually reached a level of maturity comparable to adult kidneys. These findings indicate that the primary challenge in replacing renal function thus far has been insufficient tissue volume.

With respect to life-sustaining function, while transplanted MNs with urinary tract connection demonstrated filtration function as confirmed by inulin clearance^7^ and showed the capacity to prolong survival of nephrectomized rats,^15–17^ long-term survival of anephric animals had not been achieved. We examined the hypothesis that increasing the number of transplants could accomplish this goal. To increase the number and amount of transplantable tissues, we developed a novel technique involving the fusion of immature bladder tissues and multiple anastomoses to the host ureter. This approach reconstitutes the essential urinary drainage system for multiple transplanted embryonic kidneys. Ureteric buds, primordia of the renal collecting duct and ureter, can be connected to share a common lumen by cutting them and maintaining contact between the epithelial ends.^60^ However, it was previously unclear whether this capacity is specific to the ureteric bud or can be extended to the bladder. Our results showed that the split surfaces of the bladder can be caused to fuse together *in vivo* and acquire bladder functionality through simply aligning their cross-sections and fixing them with 2 stitches. Our approach allowed for the transplantation of multiple kidney primordia while significantly reducing the number of anastomoses required during urinary tract reconstruction. Consequently, we found that the long-term survival of anephric rats required at least 20 MNs, and notably, the clearance obtained from a single MN was lower than that previously reported.^16,61,62^ This unexpected low filtration capacity revealed several challenges regarding the functional capacity of transplanted MNs for future investigation. While the combined weight and number of glomeruli in the 20 transplanted embryonic kidneys were approximately 60% of those in healthy bilateral kidneys; however, the creatinine clearance was only 15%. This may be due to thin infiltrating blood vessels, which replace physiological renal arteries, potentially reducing blood flow into the embryonic kidney and intraglomerular pressure.^6^ The immature urine-concentration ability was shown to be due to the shortness of the countercurrent system segments; this is likely attributable to their small size, highlighting the importance of organ size. While the factors determining organ size during *in vivo* growth remain unclear, early loss of progenitors in the transplanted embryonic kidneys is considered a contributing factor.^59^ Mammalian nephron progenitor cells have limited self-renewal after birth, and preterm birth results in smaller kidneys.^63^ Elucidating the mechanism of this postnatal cessation of kidney growth may lead to enlarge MNs after transplantation, increasing the number of nephrons as well as lengthening the tubular segment of individual nephrons. Further research is warranted to expand individual MNs and enhance their function per single nephron to minimize the number of MNs required for therapeutic efficacy and achieve successful organ replacement therapy using the embryonic organ transplantation approach.

This study had a few limitations. First, the precise mechanism for the fusion of embryonic bladders was not elucidated. Understanding this mechanism could allow the integration of more embryonic bladders without microsurgical anastomosis. Second, we evaluated filtration function using creatinine clearance, which is known to overestimate kidney function especially in the lower glomerular filtration rate range due to enhanced tubular secretion of creatinine.^64^ Indeed, creatinine clearance in 5/6 nephrectomy rats was approximately one-fourth of that in healthy rats, higher than the expected one-sixth. While this might reflect functional compensation through hyperfiltration, it could also represent an overestimation associated with this method. Therefore, the actual function of 20 MNs might be even lower than the measured 15% of healthy controls. Finally, this study was conducted only in a syngeneic rat model. Further evaluation of maturation capacity in allo- and xenotransplantation models considering immune responses, as well as validation of therapeutic efficacy in large animals, will be essential before proceeding toward clinical translation.

In summary, we developed a novel strategy to increase transplantable embryonic kidney tissue and successfully achieved long-term survival of anephric rats with MN transplants. Although significant efforts will be needed before clinical implementation, our results provide evidence for the functionality of transplanted embryonic organs and highlight their potential as candidates for therapeutically transplantable organs.

## Supplementary Material

Supplement Info

Supplement Video-1

Supplement Video-2

Supplement Video-3

Supplementary material is available online at www.kidney-international.org.

## Data Availability

The scRNA-seq data generated in this study have been deposited in the Gene Expression Omnibus under accession number GSE268927, at https://www.ncbi.nlm.nih.gov/geo/query/acc.cgi?acc=GSE268927. We also analyzed a publicly available dataset (Sequence Archive Read: PRJNA 649702), at https://www.ncbi.nlm.nih.gov/bioproject/PRJNA649702. All other data are available from the corresponding author on reasonable request.

## Translational Statement

To address the global organ shortage, novel transplantable kidneys need to be developed. Embryonic kidneys, or metanephroi, have not been recognized as therapeutically transplantable organs due to their inadequate functionality despite their ability to mature in the host body through tissue transplantation without vascular anastomosis. In this study, for the first time, a substantial number of metanephroi were transplanted and integrated into the host urinary tract using a novel surgical approach, demonstrating their life-supporting function and adult-equivalent maturity. This advancement significantly progresses the clinical realization of a novel renal replacement therapy using embryonic kidney tissue.
