# Supplementary material for: Life-supporting functional kidney replacement by integration of embryonic metanephros-bladder composite tissue transplants
Source: Kidney Int. Author manuscript; Available in PMC 2025 Oct 15. (PMC12521919; doi:10.1016/j.kint.2025.02.024)
Supplement: Supplement Info [file NIHMS2113639-supplement-Supplement_Info.pdf]

Supplementary Information

Life-supporting functional kidney replacement by surgical integration of embryonic metanephros-bladder composite tissue transplants

Yoshitaka Kinoshita[1,2], Eiji Kobayashi[3], Kenji Matsui[1], Yuka Inage[1,4], Keita Morimoto[1], Shutaro Yamamoto[1,5], Satomi Iwai[6], Kento Kitada[7], Kentaro Iwasawa[8], Yatsumu Saito[1], Toshinari Fujimoto[1], Kei Matsumoto[1], Shushi Nagamori[9], Akira Nishiyama[7], Haruki Kume[2], Takanori Takebe[8,10,11,12], Takashi Yokoo[1], Shuichiro Yamanaka[1]

[1]Division of Nephrology and Hypertension, Department of Internal Medicine, The Jikei University School of Medicine, Tokyo, Japan
[2]Department of Urology, Graduate School of Medicine, The University of Tokyo, Tokyo, Japan
[3]Department of Kidney Regenerative Medicine, The Jikei University School of Medicine, Tokyo, Japan
[4]Department of Pediatrics, The Jikei University School of Medicine, Tokyo, Japan
[5]Department of Urology, The Jikei University School of Medicine, Tokyo, Japan
[6]Laboratory of Small Animal Surgery 2, School of Veterinary Medicine, Kitasato University, Aomori, Japan
[7]Department of Pharmacology, Faculty of Medicine, Kagawa University, Kagawa, Japan
[8]Division of Gastroenterology, Hepatology & Nutrition, Developmental Biology and Center for Stem Cell and Organoid Medicine (CuSTOM), Cincinnati Children's Hospital Medical Center, OH, USA
[9]Center for Stable Isotope Medical Research, The Jikei University School of Medicine, Tokyo, Japan
[10]Department of Pediatrics, University of Cincinnati College of Medicine, OH, USA
[11]Premium Research Institute for Human Metaverse Medicine (WPI-PRIMe), and Department of Genome Biology, The University of Osaka, Osaka, Japan
[12]Human Biology Research Unit, Institute of Integrated Research, Institute of Science Tokyo (Science Tokyo), Tokyo, Japan

Corresponding authors

Shuichiro Yamanaka (shu.yamanaka@jikei.ac.jp) or Eiji Kobayashi (eijikoba@jikei.ac.jp)

Table of Contents

Supplementary Methods
Supplementary References

Supplementary Figures
Supplementary Fig. S1 | Protocol of refined postoperative management.
Supplementary Fig. S2 | Long-term outcome of MN graft after urinary tract reconstruction.
Supplementary Fig. S3 | Bladder urine accumulation volume after transplantation of single MNBs and integrated MNB grafts.
Supplementary Fig. S4 | Factors affecting the survival of rats transplanted with integrated MNB grafts.
Supplementary Fig. S5 | Cell type distribution in MN grafts based on scRNA-seq analysis.
Supplementary Fig. S6 | Single-cell RNA sequencing trajectory analysis of major kidney cell types.
Supplementary Fig. S7 | Gene expression of maturation-related marker for various nephron segments.

Supplementary Tables
Supplementary Table S1 | Summary of transplant conditions and postoperative management after host kidney removal.
Supplementary Table S2 | Composition of specialized diets used in the experiments.
Supplementary Table S3 | List of primary immunostaining antibodies.
Supplementary Table S4 | List of TaqMan assays.

Supplementary Videos
Supplementary Movie S1 | Contrast-enhanced computed tomography to visualize the urinary tract. The urinary tract is visualized via contrast-enhanced computed tomography (CT) of rats transplanted with an integrated MNB graft at 8 weeks post-transplantation. Axial CT images progress from the cranial to the caudal side. Scale bar, 10 mm.
Supplementary Movie S2 | Tissue clearing to visualize the vasculature of the transplanted embryonic kidneys. The vasculature of the five-integrated MNB graft-transplanted rat is visualized by perfusing fluorescently labelled lectin 13 weeks post-transplantation. Vascular invasion into the grafts and glomeruli within the grafts is observed. The video progresses from the dorsal side (the host abdominal aorta could be observed) to the ventral side (five transplanted integrated MNB grafts could be observed). Scale bar, 5mm.
Supplementary Movie S3 | High-magnification view of vascular networks in cleared MN grafts and adult control kidneys. MN grafts at 13 weeks post-transplantation (left)

demonstrate well-developed peritubular and glomerular capillaries comparable to those in 8-week healthy control kidneys (right). Scale bar, 100 μm.

Supplementary Methods

Anesthesia and postoperative care of animals

All surgical procedures were performed using a surgical microscope (S9D or M205FA, Leica) under general anesthesia with isoflurane (induction at 5%, maintenance at 2%), analgesia through subcutaneous administration of butorphanol (1.0 mg/kg), and enrofloxacin (10 mg/kg).

5/6 nephrectomy

A two-step 5/6 nephrectomy model served as a control mimicking chronic kidney disease.[1] Eight-week-old LEW rats underwent surgical removal of the upper and lower thirds of the left kidney through a flank incision. No vascular clamping was performed, and hemostasis was achieved only by digital compression. One week later, the right kidney was removed through a contralateral flank incision. The animals were analyzed 1–2 weeks after the right nephrectomy.

Contrast-enhanced CT

Rats that underwent transplantation and reconstruction with an integrated MNB graft were subjected to contrast-enhanced CT at 8 weeks post-transplantation to confirm the integrity of the urinary tract connection. After anesthesia with subcutaneous administration of 0.375 mg/kg medetomidine, 2 mg/kg midazolam, and 2.5 mg/kg butorphanol, a CT image was acquired using a rodent CT scanner (LaTheta LCT-200, ALOKA) 150 min after intravenous administration of 0.5 mL of a contrast agent (Iopamilon, Bayer) diluted with 0.5 mL saline. The scanning parameters included a pixel size, slice thickness, and slice spacing of 120 µm, low X-ray tube voltage, artifact elimination for soft tissues, high scanning resolution, and high-precision rotation speed. Subsequently, a region of interest was manually set in the retroperitoneal cavity using ImageJ software,[2] and the 3D Viewer plugin was used to construct a three-dimensional image of the high-density area to visualize the urinary tract.

Blood, urine, and tissue processing and histological evaluation

Blood samples were obtained from the tail vein or inferior vena cava via laparotomy after a 30–60 min fasting period. Whole blood samples were processed using an portable blood analyzer (iSTAT, Abbott), whereas serum samples were analyzed using a chemistry analyzer (DRI-CHEM NX-600, Fujifilm) or submitted to external clinical laboratories (SRL). Urine samples collected from metabolic cages were submitted to SRL for processing. Tissue samples were either fixed with 10% neutral buffered formalin and embedded in paraffin or fixed with 4% paraformaldehyde, dehydrated in 10% and 20% sucrose solutions, and embedded in an optimal cutting temperature (OCT) compound for frozen sectioning.

Paraffin-embedded sections measuring 5 μm in thickness or 10-μm-thick OCT frozen sections were stained using standard protocols, followed by imaging using the digital microscope (BZ-X800, Keyence) or used for subsequent immunohistochemistry analyses.

Immunohistochemistry

For immunohistochemistry, primary antibodies used were demonstrated in Supplementary Table S3. Antigen retrieval was performed via incubation in pH 6.0 citrate buffer at 121 °C for 10 min for paraffin sections and antigen retriecal solution (Histo VT One, Nacalai Tesque) at 70 °C for 20 min for frozen sections. Endogenous peroxidase was blocked with 0.3% hydrogen peroxide in methanol for 20 min at room temperature for horseradish peroxidase (HRP) staining. After blocking with blocking reagent (Blocking One Histo, Nacalai Tesque) for 10 min at room temperature, the sections were incubated with primary antibodies and subsequently stained with HRP-conjugated anti-goat or anti-rabbit secondary antibodies (Histofine Simple Stain, Nichirei Biosciences). Fluorescent staining was achieved using AlexaFluor488-, AlexaFluor546-, or AlexaFluor647-conjugated anti-rabbit, anti-mouse, anti-goat, or anti-guinea pig secondary antibodies (1:200; Thermo Fisher Scientific). Nuclei were counterstained with 4',6-diamidino-2-phenylindole. The sections were mounted with mounting medium (ProLong Gold Antifade Mountant, Thermo Fisher Scientific) and examined under a confocal laser scanning microscope (LSM880, ZEISS).

Electron microscopy

Tissue samples were perfusion-fixed with 10% neutral buffered formalin and processed as outlined below. For transmission electron microscopy, specimens were fixed with 2% glutaraldehyde in 0.1 M phosphate buffer (pH 7.3) overnight at 4 °C, followed by post-fixation with 1% osmium tetroxide in the same buffer at 4 °C for 2 h. Dehydration was conducted using a graded series of ethanol, after which the specimens were placed in propylene oxide and subsequently embedded in epoxy resin (Epok 812, Okenshoji). Next, 60-nm-thick ultrathin sections were prepared using a diamond knife, stained with uranium acetate and lead citrate, and observed under a transmission electron microscope (JEM-1400Plus, JEOL) at 100 kV. For scanning electron microscopy, samples were fixed in 1.2% glutaraldehyde in 0.1 M phosphate buffer (pH 7.3) overnight at 4 °C and dehydrated in a graded ethanol series, followed by critical point drying (Hitachi). After coating with osmium tetroxide using an osmium coater (Vacuum Device), the samples were observed under a scanning electron microscope (Regulus 8100, Hitachi) at 2.0 kV.

Quantitative RT-PCR

To investigate the expression of maturation-related genes, total RNA was extracted using the RNA extraction kit (RNeasy Plus Mini, Qiagen) and reverse-transcribed to cDNA using

reverse transcription kit (PrimeScript™ RT Reagent Kit with gDNA Eraser, Qiagen), according to the manufacturer's instructions. Real-time quantitative PCR was performed using a real-time PCR system (Rotor-Gene Q, Qiagen) with probe-based qPCR reagents (TaqMan™ Gene Expression Assays and TaqMan™ Gene Expression Master Mix, Applied Biosystems). All samples were analyzed in duplicate using two-standard curve quantification method in Rotor-Gene Q software. Gene expression levels were calculated normalized to glyceraldehyde-3-phosphate dehydrogenase (*Gapdh*) as a housekeeping gene, relative to one of the E16 control samples. The TaqMan assays used in this study are listed in Supplementary Table 4.

scRNA-seq of transplanted embryonic kidneys

Rats transplanted with five integrated MNB grafts were euthanized under anesthesia 3 weeks after bilateral nephrectomy (16 weeks post-transplantation). Parenchyma from the transplanted kidney was collected, minced into millimeter-sized pieces, and thoroughly mixed. Approximately 100 mg of the sample was digested with 1 mL of dissociation reagent (Accutase, Innovative Cell Technologies) at 37 °C for 15 min and gently pipetted, followed by neutralization of enzymatic activity with 1 mL of minimum essential medium α (Gibco) with 20% fetal bovine serum (FBS) and 0.4% penicillin/streptomycin. Cell suspensions were filtered through a 35 μm nylon cell strainer and centrifuged at 400 ×*g* for 5 min, after which the supernatant was discarded. Following debris removal using a debris removal solution (Miltenyi Biotec) according to the manufacturer's protocol, the cell pellets were washed three times with Dulbecco's phosphate-buffered saline (DPBS)+10% FBS. Finally, the cells were resuspended in an appropriate volume of DPBS+10% FBS after confirmation of viable cell abundance through cell counts. The cells were loaded onto a single-cell isolation system (Chromium Single-Cell Instrument, 10x Genomics) targeting 10,000 cells to generate single-cell gel bead-in-emulsions (GEMs). Subsequently, scRNA-seq libraries were prepared using the library preparation kit (Chromium Next GEM Single Cell 3′ GEM Kit v3.1, 10x Genomics). GEM reverse transcription (GEM-RT) was performed to produce a barcoded, full-length cDNA from polyadenylated mRNA. After incubation, the GEMs were broken, the pooled post-GEM-RT reaction mixtures were recovered, and cDNA was purified using silane magnetic beads (DynaBeads MyOne Silane Beads, PN37002D, Thermo Fisher Scientific). The purified post-GEM-RT product was amplified via PCR. Sequencing libraries were prepared following the manufacturer's instructions. Sequencing was performed in paired-end mode (read1: 28 bp; read2: 90 bp) using a high-throughput sequencer (DNBSEQ-G400 sequencer, MGI Tech), resulting in more than 420 million reads per sample.

scRNA-seq data processing

scRNA-seq reads were processed using the CellRanger 7.1.0 pipeline against the Rnor_6.0-104 reference genome in 3′ v3 chemistry mode (10x Genomics). Cells were filtered based on a minimum threshold of 200 detected genes and a minimum unique molecular identifier count of 1,000 per cell. Further refinement involved the removal of cells expressing more than 20% mitochondrial genes and more than 15% ribosomal genes. Normalization procedures encompassed regression variables accounting for mitochondrial content and total RNA counts, which were adjusted for using scTransform (Seurat version 4.4).[3] Anchor-based sample integration was conducted without scaling owing to prior normalization. Principal component analysis (PCA) based on variable genes was conducted, followed by the construction of a shared nearest neighbor graph for cell clustering. Integration across different conditions was performed using an anchor-based reciprocal PCA method.[3] Data visualization was facilitated through uniform manifold approximation and projection.

Downstream analysis of scRNA-seq

After unsupervised clustering of all filtered cells, differentially expressed gene (DEG) analysis was conducted to identify marker genes for each cell cluster using the FindAllMarkers function in Seurat. Cell types were annotated based on the expressions of established marker genes.[4,5,6] Single-cell pseudotime analysis was employed in conjunction with DEG analysis to explore developmental transitions across different sample conditions. Pseudotime trajectory analysis, executed using Monocle 2.0, utilized the kidney day 0 cell population as the root population to delineate cellular trajectories over time.[7,8] Subsequently, BEAM analysis was conducted to profile gene transitions along each trajectory. The genes visualized in the BEAM were further narrowed down by considering the DEGs in each cluster of Monocle derived by density peak clustering. The number of clusters was manually adjusted so that at least one cluster in the BEAM heatmap could cluster upregulated genes in each cell fate. Gene Ontology enrichment analysis using all DEGs was performed using Metascape[9] with the Kyoto Encyclopedia of Genes and Genomes pathway.

Tissue clearing for visualization of the vasculature

Lycopersicon lectin, DyLight 649 (DL-1178, Vector Laboratories) was administered intravenously at a dosage of 2 μg/g BW prior to perfusion fixation, with a time interval of 10 min. Tissue samples, including 8-week-old healthy rat kidneys or five integrated MNB grafts 13 weeks post-transplantation, were collected for analysis. Samples were subjected to dehydration in methanol for over 48 h, followed by 48-h delipidation with dichloromethane (Sigma) and re-immersion in methanol. Refractive index matching with benzyl alcohol/benzyl benzoate was performed, and cleared samples were imaged using a light-sheet microscope (LCS SPIM, Bruker) and confocal spinning disk microscope (CSU-W1 SoRa, Yokogawa). Glomerular quantification was conducted using the 'spot' function in Imaris

software (Bitplane), which identified glomeruli based on their distinct spherical structure attributed to lectin accumulation.

Supplementary References

S1. Uchiyama T, Tatsumi N, Kamejima S, et al. Hypermethylation of the CaSR and VDR genes in the parathyroid glands in chronic kidney disease rats with high-phosphate diet. *Hum Cell.* 2016;29(4):155–61. doi: 10.1007/s13577-016-0143-9

S2. Schindelin J, Arganda-Carreras I, Frise E, et al. Fiji: an open-source platform for biological-image analysis. *Nat Methods*. 2012;9(7):676–682. doi:10.1038/nmeth.2019

S3. Stuart T, Satija R. Integrative single-cell analysis. *Nat Rev Genet*. 2019;20(5):257–272. doi:10.1038/s41576-019-0093-7

S4. Park J, Shrestha R, Qiu C, et al. Single-cell transcriptomics of the mouse kidney reveals potential cellular targets of kidney disease. *Science*. 2018;360(6390):758–763. doi:10.1126/science.aar2131

S5. Lu Y-A, Liao C-T, Raybould R, et al. Single-nucleus RNA sequencing identifies new classes of proximal tubular epithelial cells in kidney fibrosis. *J Am Soc Nephrol*. 2021;32(10):2501–2516. doi:10.1681/ASN.2020081143

S6. Liu W-B, Huang G-R, Liu B-L, et al. Single cell landscape of parietal epithelial cells in healthy and diseased states. *Kidney Int*. 2023;104(1):108–123. doi:10.1016/j.kint.2023.03.036

S7. Trapnell C, Cacchiarelli D, Grimsby J, et al. The dynamics and regulators of cell fate decisions are revealed by pseudotemporal ordering of single cells. *Nat Biotechnol*. 2014;32(4):381–386. doi:10.1038/nbt.2859

S8. Qiu X, Hill A, Packer J, et al. Single-cell mRNA quantification and differential analysis with Census. *Nat Methods*. 2017;14(3):309–315. doi:10.1038/nmeth.4150

S9. Zhou Y, Zhou B, Pache L, et al. Metascape provides a biologist-oriented resource for the analysis of systems-level datasets. *Nat Commun*. 2019;10(1):1523. doi:10.1038/s41467-019-09234-6

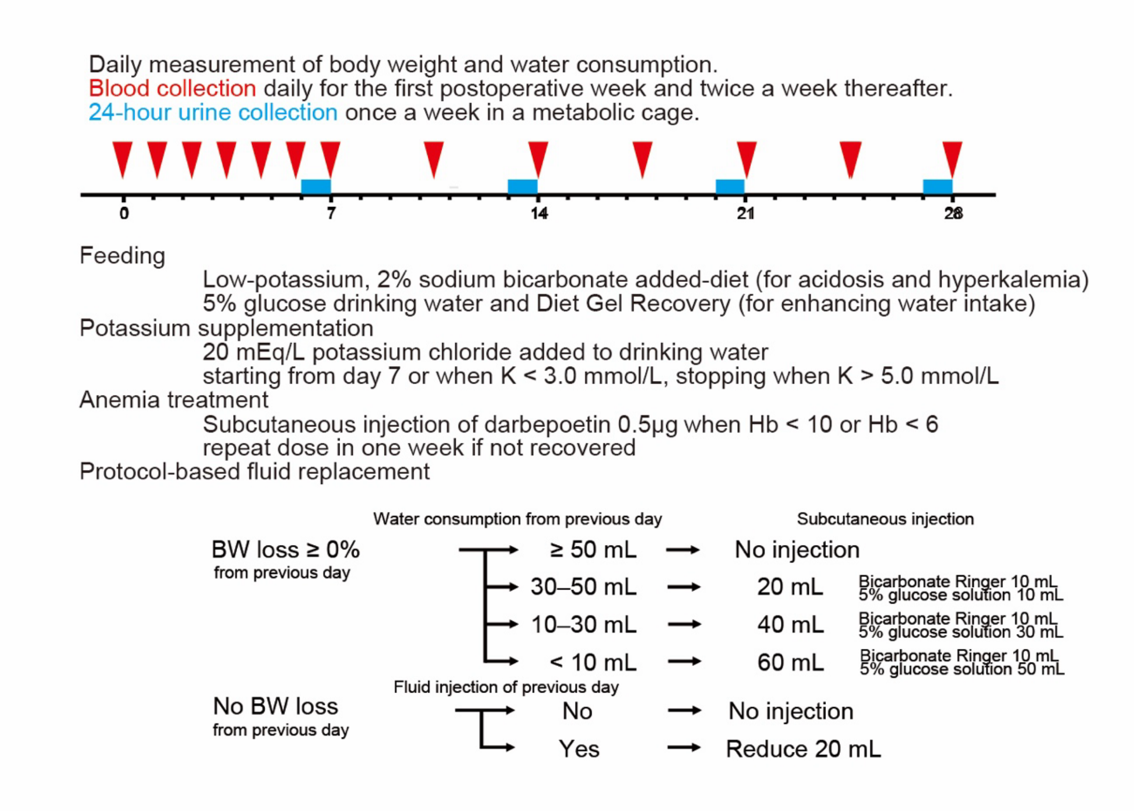

Supplementary Fig. S1 | Protocol of refined postoperative management. Schedule for observations and tests, as well as protocols for postoperative support, are shown.

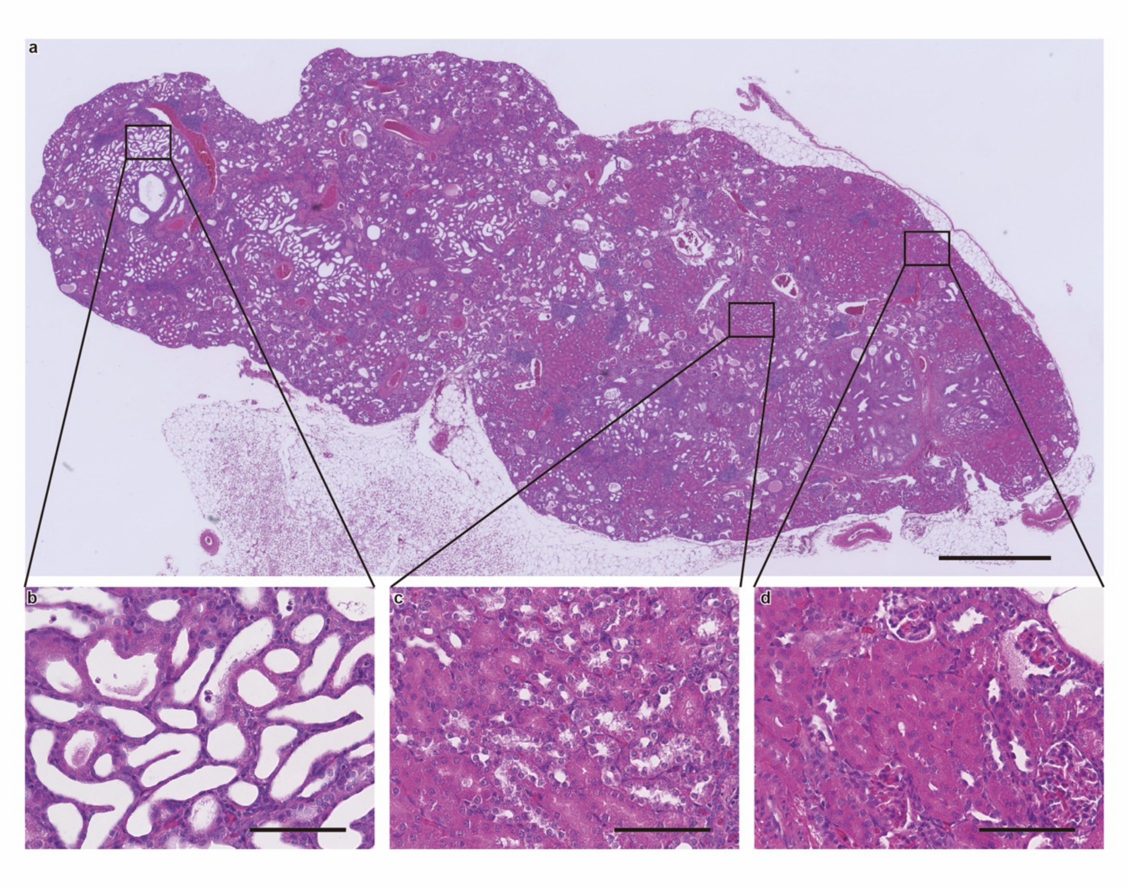

Supplementary Fig. S2 | Long-term outcome of MN graft after urinary tract reconstruction. Hematoxylin and eosin staining of MN component from MNB transplanted in syngeneic rats at 13 weeks after urinary tract reconstruction (16 weeks post-transplantation). **(a)** Global appearance. Scale bar, 1 mm. **(b–d)** Higher magnification images showing slightly dilated tubules **(b)**, and medullar **(c)** and cortical **(d)** regions with intact histological features. Scale bars, 100 μm.

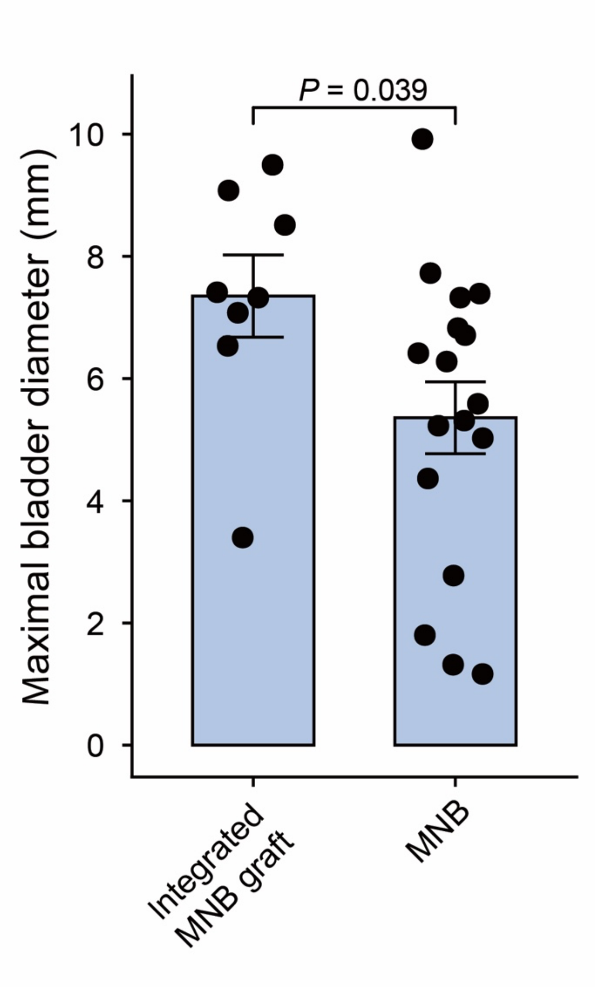

Supplementary Fig. S3 | Bladder urine accumulation volume after transplantation of single MNBs and integrated MNB grafts. Maximum diameter of urine accumulation measured by transabdominal ultrasonography at 3 weeks post-transplantation. The increased bladder size observed in the integrated MNB grafts likely reflects enhanced urine accumulation due to the increased number of metanephric tissues.

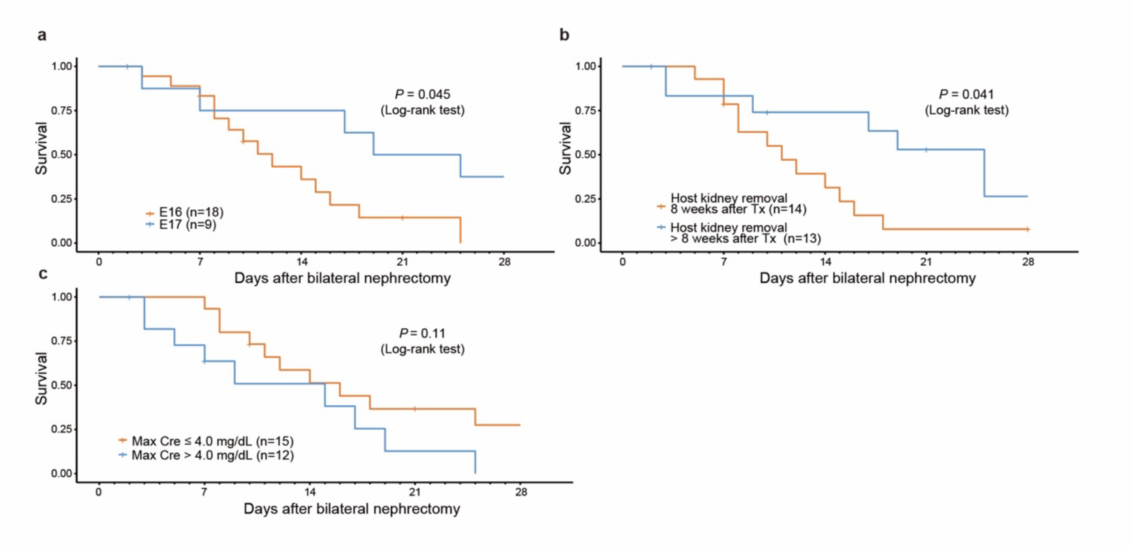

Supplementary Fig. S4 | Factors affecting the survival of rats transplanted with integrated MNB grafts. Kaplan–Meier curves showing the survival of groups (**a**) transplanted with E16 and E17 integrated MNB grafts, (**b**) undergoing host kidney removal 8 weeks and more than 8 weeks post-transplantation, and (**c**) with peak serum creatinine levels ≤ 4.0 mg/dL and > 4.0 mg/dL. Euthanasia for sample collection and deaths attributed to technical errors during anesthesia has been censored. Cre, creatinine; MNB, metanephros–bladder composite.

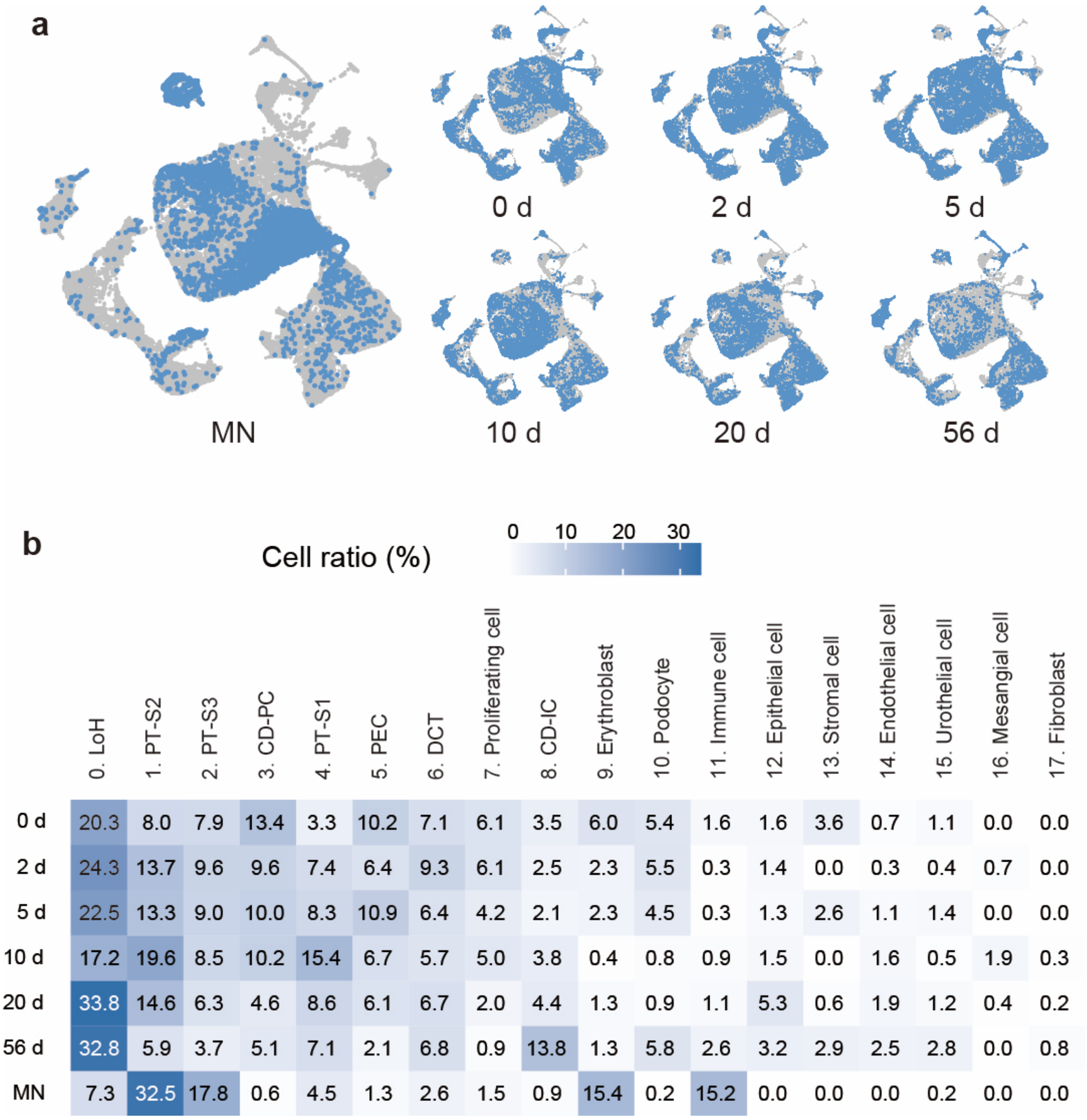

Supplementary Fig. S5 | Cell type distribution in MN grafts based on scRNA-seq analysis. (a) UMAP visualization showing distribution of cells from MN grafts at 16 weeks post-transplantation and healthy control kidneys at postnatal days 0, 2, 5, 10, 20, and 56. **(b)** Heatmap depicting relative abundance of each cell type across samples. MN shows lower proportions of LoH, CD-PC, DCT, CD-IC, and podocytes compared to control kidneys. CD-IC, intercalated cell of the collecting duct; CD-PC, principal cell of the collecting duct; DCT, distal convoluted tubule; LoH, Loop of Henle; MN, metanephros; MNB, metanephros-bladder composite; PEC, glomerular parietal epithelial cell; PT-S1, proximal tubule segment 1; PT-S2, proximal tubule segment 2; PT-S3, proximal tubule segment 3; scRNA-seq, single-cell RNA sequencing; UMAP, uniform manifold approximation and projection.

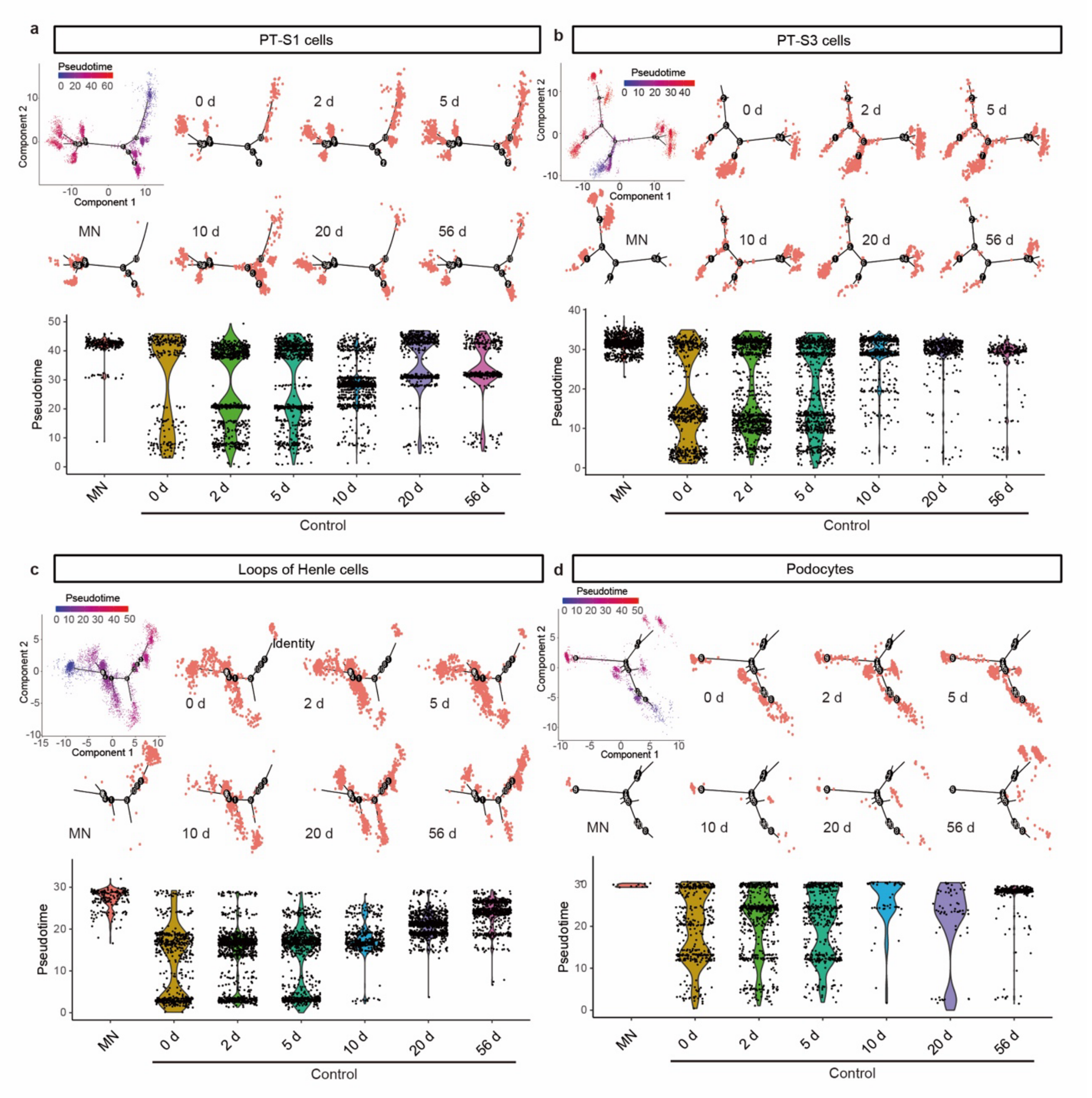

Supplementary Fig. S6 | Single-cell RNA sequencing trajectory analysis of major kidney cell types. scRNA-seq data from transplanted MNs sampled 3 weeks after bilateral nephrectomy (16 weeks after integrated MNB transplantation) were analyzed after integration with scRNA-seq data obtained from kidney samples at postnatal days 0, 2, 5, 10, 20, and 56. Monocle 2-based trajectory analysis of (**a**) PT-S1, (**b**) PT-S3, (**c**) LoH, and (**d**) podocytes is shown. In each panel, upper plots show cells projected onto tSNE space and colored by predicted pseudotime, and lower plots show violin plots of pseudotime distribution in MN samples and healthy controls at various ages (P0–P56). Most cells derived from the MN samples show advanced pseudotime. LoH, Loop of Henle; MN, metanephros; MNB, metanephros-bladder composite; PT-S1, proximal tubule segment 1; PT-S3, proximal tubule segment 3; scRNA-seq, single-cell RNA sequencing; t-SNE, t-distributed stochastic neighbor embedding.

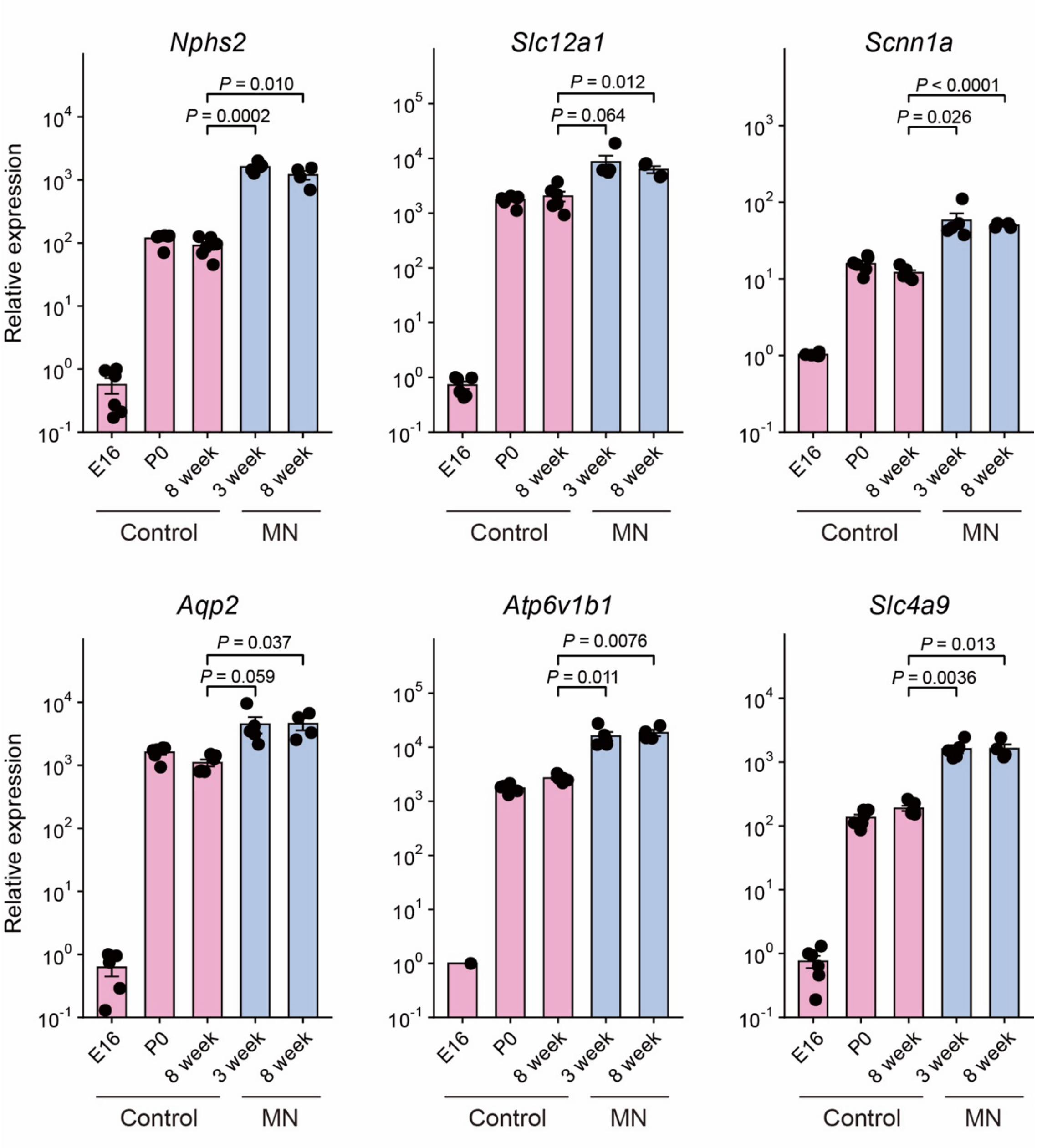

Supplementary Fig. S7 | Gene expression of maturation-related marker for various nephron segments. qRT-PCR analysis of maturation-related markers in each nephron segment. Gene expression was compared among E16, P0, and 8-week control kidneys, and MNs at 3 weeks post-transplantation (without urinary tract reconstruction) and 8 weeks post-transplantation (5 weeks after urinary tract reconstruction). Expression values are normalized to *Gapdh* and shown relative to one of the E16 control samples. Data are means ± s.e.m. Points represent biological replicates from at least four independent experiments. Statistical significance was determined by two-tailed Student's unpaired t-tests. MN, metanephros

Supplementary Table S1 | Summary of transplant conditions and postoperative management after host kidney removal.

			Transplant condition					Postoperative management after host kidney removal				
Rat ID	Survival (day)	Peak serum creatinine (mg/dL)	Embryonic day of donor fetus	Number of metanephroi	Number of integrated MNB grafts	Day of urinary reconstruction	Weeks of host kidney removal	Diet	Acidodis treatment (oral gavage)	Potassium supplementation (added to drinking water)	Anemia treatment (darbeportin subcutaneous injection)	Fluid therapy (subcutaneous injection)
220610	3	8.2	E17	12	3	21	10.3	low-K				Bicarbonate Ringer + 5% glucose[c]
220617	28	3.9	E17	20	5	23	9.7	low-K			2.5μg[c]	Bicarbonate Ringer + 5% glucose[c]
220701-1	17	5.8	E17	16	4	24	9.9	low-K			2.5μg[c]	Bicarbonate Ringer + 5% glucose[c]
220701-2	25	6.2	E17	16	4	21	14.3	low-K	$NaHCO_3$[c]		1.0μg[c]	Bicarbonate Ringer + 5% glucose[c]
220714	7	6.2	E16	16	4	21	12.4	low-K	$NaHCO_3$[c]		1.0μg[c]	Bicarbonate Ringer + 5% glucose[c]
220728	3	6.1	E16	12	3	22	13.6	low-K	$NaHCO_3$[c]			Bicarbonate Ringer + 5% glucose[c]
220811	25	3.7	E16	16	4	21	12.4	low-K	$NaHCO_3$[c]	10–20mmol/L[c]	1.0μg[c]	Bicarbonate Ringer + 5% glucose[c]
220909	7	2.9	E17	20	6 transplanted (5 reconstructed)	24	8.9	low-K	$NaHCO_3$[c]	10–20mmol/L[c]		Bicarbonate Ringer + 5% glucose[c]
220916	19	5.3	E17	16	4	23	9.9	low-K	$NaHCO_3$[c]	10–20mmol/L[c]	1.0μg[c]	Bicarbonate Ringer + 5% glucose[c]
220923	21[a]	2.7	E16	20	5	21	13.4	low-K	$NaHCO_3$[c]	10–20mmol/L[c]	1.0μg[c]	Bicarbonate Ringer + 5% glucose[c]
221104	15	5.1	E16	20	5	27	8.7	low-K	$NaHCO_3$[c]	10–20mmol/L[c]	1.0μg[c]	Bicarbonate Ringer + 5% glucose[c]
221113	28	3.2	E17	20	5	22	8.1	low-K→ low-K, $NaHCO_3$		10–20mmol/L[c]	1.0μg[c]	Bicarbonate Ringer + 5% glucose[c]
221120	28	3.5	E17	20	5	22	11.6	low-K, $NaHCO_3$		10–20mmol/L[c]	1.0μg[d]	Bicarbonate Ringer + 5% glucose[c]
221208	10[b]	2.7	E16	16	4	21	9.7	low-K, $NaHCO_3$		Protocol	1.0μg[d]	Bicarbonate Ringer + 5% glucose[c]
221218	2[b]	5.1	E17	16	4	22	10.1	low-K, $NaHCO_3$		Protocol	1.0μg[d]	Protocol
221223	9	4.3	E16	16	4	24	9.4	low-K, $NaHCO_3$		Protocol	1.0μg[d]	Protocol
230106	11	3.1	E16	16	4	21	8.6	low-K, $NaHCO_3$		Protocol	1.0μg[d]	Protocol
230203	16	2.7	E16	16	4	21	8.0	low-K, $NaHCO_3$		Protocol	1.0μg[d]	Protocol
230210	8	2.8	E16	16	4	21	8.0	low-K, $NaHCO_3$		Protocol	1.0μg[d]	Protocol
230309	18	2.9	E16	16	4	21	8.1	low-K, $NaHCO_3$		Protocol	1.0μg[d]	Protocol
230316	5	8.0	E16	16	4	29	8.9	low-K, $NaHCO_3$		Protocol	1.0μg[d]	Protocol
230330	8	2.7	E16	16	4	21	8.9	low-K, $NaHCO_3$		Protocol	1.0μg[d]	Protocol
230406	7	4.2	E16	16	4	21	8.9	low-K, $NaHCO_3$		Protocol	1.0μg[c]	Protocol
230413	12	3.0	E16	16	4	21	8.9	low-K, $NaHCO_3$		Protocol	0.5μg[d]	Protocol
230427	10	3.8	E16	16	4	21	8.9	low-K, $NaHCO_3$		Protocol	Protocol	Protocol
230518	14	3.1	E16	16	4	21	8.9	low-K, $NaHCO_3$		Protocol	Protocol	Protocol
230615	7[a]	4.1	E16	16	4	21	8.6	low-K, $NaHCO_3$		Protocol	Protocol	Protocol

After exhaustive trial and error, the protocol in Supplementary Fig. S1 was finalized (highlighted in grey). The diet compositions (low-potassium diet and low-potassium, 2% sodium bicarbonate-supplemented diet) are detailed in Supplementary Table S2. For oral gavage acidosis correction, 8.4% (w/v) sodium bicarbonate solution was administered. Potassium supplementation was provided using a 1 mEq/mL potassium chloride solution. For fluid therapy, a combination of bicarbonate Ringer's solution and 5% glucose solution was used.

[a] Euthanized for sample collection.

[b] Death attributed to technical errors during anesthesia.

[c] Dosage and administration intervals were adjusted at the discretion of the observer.

[d] Darbepoetin was administered on days 0, 7, 14, 21, and 28 and when hemoglobin < 10.0 g/dL.

Supplementary Table S2| Composition of specialized diets used in the experiments.

a

Ingredients in 100 g of pelleted diet (g)	
Corn starch	45.5
Milk casein	24.5
Granulated sugar	10.0
Mineral Mixture (A)	7.0
Corn oil	6.0
Crystalline cellulose	3.0
Cellulose powder	2.0
Gelatinized starch	1.0
Vitamin Mixture (B)	1.0
Total	100.0

(A) Mineral mixture (mg)	
$CaHPO_4 \cdot 2H_20$	3687.50
Corn Starch	1614.90
$MgSO_4 \cdot 7H_20$	800.00
NaCl	600.00
$FeC_6H_5O_7 \cdot nH_2O$	190.00
$CaCO_3$	83.00
$MnSO_4 \cdot 5H_2O$	15.40
$2ZnCO_3 \cdot 3Zn(OH)_2 \cdot H_20$	6.00
$Ca(IO_3)_2$	1.54
$CuSO_4 \cdot 5H_2O$	1.26
$CoCl_2 \cdot 6H_2O$	0.40
Total	7000.00

(B) Vitamin mixture (mg)	
Corn starch	635.378
Choline chloride	300.000
Vitamin E(50%)	20.000
Inositol (99%)	15.000
Nicotinic acid	10.150
Para-aminobenzoic acid (100%)	10.150
Calcium D-Pantothenate	2.000
Vitamin B2 (80%)	1.872
Vitamin B1	1.500
Vitamin A (1000000 IU/g)	1.200
Vitamin B6	1.020
Biotin (2%)	0.500
Vitamin D3 (500000 IU/g)	0.480
Vitamin K3	0.300
Vitamin B12 (2%)	0.250
Folic acid	0.200
Total	1000.000

b

Ingredients in 100 g of pelleted diet (g)	
Corn starch	30.2
Milk casein	24.5
Gelatinized corn starch	14.0
Caster sugar	10.0
Mineral Mixture (A)	7.0
Corn oil	6.0
Crystalline cellulose	3.0
Cellulose powder	2.0
Vitamin Mixture (B)	1.0
Choline chloride	0.3
Sodium bicarbonate	2.0
Total	100.0

(A) Mineral mixture (mg)	
$CaHPO_4$	2,918.000
Corn starch	2,794.997
NaCl	600.000
$MgSO_4$	390.700
$FeC_6H_5O_7 \cdot nH_2O$	190.000
$CaCO_3$	80.900
$MnSO_4 \cdot 5H_2O$	15.400
$ZnCO_3$	6.663
KIO_3	1.680
$CuSO_4 \cdot 5H_2O$	1.260
$CoCl_2 \cdot 6H_2O$	0.400
Total	7,000.000

(B) Vitamin mixture (mg)	
Corn starch	927.401
Vitamin E (50%)	20.000
Inositol (99%)	14.697
Nicotinic acid	10.254
Para-aminobenzoic acid (100%)	9.998
Vitamin B12 (0.1%)	5.000
Vitamin A (325000 IU/g)	3.692
Vitamin D3 (100000 IU/g)	2.400
Calcium D-Pantothenate	2.000
Vitamin B2 (98%)	1.528
Vitamin B1	1.500
Vitamin B6	1.020
Vitamin K3	0.300
Folic acid	0.200
D-Biotin (100%)	0.010
Total	1000.000

Compositions of (**a**) the low-potassium diet and (**b**) the low-potassium, 2% sodium bicarbonate-supplemented diet.

Supplementary Table S3 | List of primary immunostaining antibodies.

Name	Host animal	Vendor	Catalog number	Dilution
GFP	Chicken	Abcam	Ab13970	1:100
UPK3	Mouse	Abcam	Ab78196	1:100
NEPHRIN	Guinea pig	Progen	GP-N2	1:100
CD31	Goat	R & D systems	AF3628	1:100
PDGFRB	Rabbit	Abcam	Ab32570	1:100
GATA3	Goat	R & D systems	AF2605	1:100
AQP2	Mouse	Santa Cruz	sc-515770	1:100
V-ATPaseB1	Rabbit	Abcam	Ab192612	1:100
LTL	Biotinylated	Vector	B-1325	1:200
E-cadherin	Mouse	BD Biosciences	610181	1:100
NKCC2	Rabbit	StressMarq Biosciences	SPC-401D	1:100
AQP1	Mouse	Santa Cruz	sc25287	1:100
RENIN	Goat	R & D systems	AF4277	1:100
CYP27B1	Rabbit	Abcam	Ab206655	1:100
AQP3	Rabbit	Sigma Aldrich	A0303	1:100
AQP4	Mouse	Abcam	Ab9512	1:100
pS256-AQP2	Rabbit	Abcam	Ab111346	1:100

Supplementary Table S4 | List of TaqMan Assays.

Gene	Assay ID	Species	Supplier
Gapdh	Rn01775763_g1	Rat	Applied Biosystems
Nphs1	Rn00674268_m1	Rat	Applied Biosystems
Nphs2	Rn00709834_m1	Rat	Applied Biosystems
Lrp2	Rn00578067_m1	Rat	Applied Biosystems
Aqp1	Rn00562834_m1	Rat	Applied Biosystems
Slc12a1	Rn00692576_m1	Rat	Applied Biosystems
Scnn1a	Rn00580652_m1	Rat	Applied Biosystems
Pecam1	Rn01467262_m1	Rat	Applied Biosystems
Kdr	Rn00564986_m1	Rat	Applied Biosystems
Tek	Rn01433346_m1	Rat	Applied Biosystems
Aqp2	Rn00563755_m1	Rat	Applied Biosystems
Pdgfrb	Rn01491838_m1	Rat	Applied Biosystems
Avpr2	Rn00569508_g1	Rat	Applied Biosystems
Atp6v1b1	Rn01765558_m1	Rat	Applied Biosystems
Slc4a9	Rn00686681_m1	Rat	Applied Biosystems
Car2	Rn01462065_m1	Rat	Applied Biosystems
